# Global analysis of common bean multidrug and toxic compound extrusion transporters (PvMATEs): PvMATE8 and pinto bean seed coat darkening

**DOI:** 10.3389/fpls.2022.1046597

**Published:** 2022-11-10

**Authors:** Nishat S. Islam, Kishor Duwadi, Ling Chen, Aga Pajak, Tim McDowell, Frédéric Marsolais, Sangeeta Dhaubhadel

**Affiliations:** ^1^ London Research and Development Centre, Agriculture and Agri-Food Canada, London, ON, Canada; ^2^ Department of Biology, University of Western Ontario, London, ON, Canada

**Keywords:** common bean, proanthocyanidin transporter, seed coat darkening, MATE (multidrug and toxic compound extrusion), epicatechin 3’-O-glucoside

## Abstract

In common bean (*Phaseolus vulgaris* L.), postharvest seed coat darkening is an undesirable trait that affects crop value. The increased accumulation of proanthocyanidins (PAs) in the seed coat results in darker seeds in many market classes of colored beans after harvest. The precursors of PAs are synthesized in the cytoplasm, and subsequently get glycosylated and then transported to the vacuoles where polymerization occurs. Thus, vacuolar transporters play an important role in the accumulation of PAs. Here, we report that common bean genome contains 59 multidrug and toxic compound extrusion genes (*PvMATE*s). Phylogenetic analysis of putative PvMATEs with functionally characterized MATEs from other plant species categorized them into substrate-specific clades. Our data demonstrate that a vacuolar transporter *PvMATE8* is expressed at a higher level in the pinto bean cultivar CDC Pintium (regular darkening) compared to 1533-15 (slow darkening). PvMATE8 localizes in the vacuolar membrane and rescues the PA deficient (*tt12*) mutant phenotype in *Arabidopsis thaliana*. Analysis of PA monomers in transgenic seeds together with wild-type and mutants suggests a possible feedback regulation of PA biosynthesis and accumulation. Identification of PvMATE8 will help better understand the mechanism of PA accumulation in common bean.

## Introduction

Plant transporters play a critical role in various cellular and biological processes and gained enormous research attention in recent years (Gani et al., 2021). They are involved in the transport of primary and specialized metabolites, organic acids, metal ions and cytotoxic substances. Several transporter families have been discovered that control the in-and-out flow of structurally diverse compounds in cellular organelles and play a major role in plant growth and development, homeostasis, reproduction and defense. Among the identified plant transporter families, major classes are- ATP-binding cassette (ABC), major facilitator superfamily (MFS), drug metabolite transporter (DMT), resistance-nodulation-division (RND) and the multidrug and toxic compound extrusion (MATE) [Reviewed in Putman et al. (2000); Yazaki (2005) and Saier and Paulsen (2001)].

The MATE gene family is widely distributed in all domains of life. Plants contain an incredibly large number of MATE transporters compared to other eukaryotes and prokaryotes (Omote et al., 2006). For example, 56 *MATE* genes are found in *Arabidopsis thaliana* (Yazaki, 2005) whereas there are 53 present in *Medicago truncatula* (Wang et al., 2017), 45 in *Oryza sativa* (Tiwari et al., 2014; Wang et al., 2016) and 117 in *Glycine max* (Liu et al., 2016), indicating a possible evolution of transporter dependence in plants due to their sessile nature. Crystal structure of MATE proteins have been elucidated for, Arabidopsis AtDTX14 for cationic substance (Miyauchi et al., 2017), *Camelina sativa* CasMATE for catechin (Tanaka et al., 2013) and *Nicotiana tabacum* NtMATE2 for nicotine (Tanaka et al., 2021) transport. All 3 of these identified crystal structures contain a V-shaped architecture with 12 transmembrane helices. In general, plant MATEs contain 400-700 amino acids with 10-12 transmembrane helices that contain 40% sequence identities within the helices (Li et al., 2002). MATEs cover a wide range of biological processes, including the transport of specialized metabolites, such as alkaloids, flavonoids and polyphenols (Yazaki, 2005; Gani et al., 2021); detoxification of heavy metals (Li et al., 2002), translocation of metal ions (Al^3+^, Fe^2+^ etc.) (Pineau et al., 2012; Wu et al., 2014; Min et al., 2019) and efflux of hormones, such as auxin, abscisic acid (ABA), salicylic acid (SA) (Zhang et al., 2014). Members of the MATE family proteins transport substrates utilizing an electrochemical gradient of Na^+^/H^+^ across the localized membrane and ATP as an energy source to drive the transport of substances in opposite directions (Saier and Paulsen, 2001; Takanashi et al., 2014).

Common bean (*Phaseolus vulgaris* L.) is the most cultivated legume crop worldwide and a rich source of flavonoids (Brigide et al., 2014). Proanthocyanidins (PAs) in the seed coat of beans serve as a source of antioxidants and contribute to their color and appearance in different market classes (McClean et al., 2002). Several studies have demonstrated the association of PAs with postharvest seed coat darkening in pinto, carioca and cranberry beans (Beninger et al., 2005; Elsadr et al., 2015; Duwadi et al., 2018; Erfatpour and Pauls, 2020; Islam et al., 2020). During storage, when exposed to environmental factors like elevated temperature, humidity and lights, beans with higher PA levels become darker in a time dependent manner. Consumers tend to show a decreased preference for the darker beans, resulting in a drop in their market value (Beninger et al., 2005; Junk-Knievel et al., 2007; Elsadr et al., 2015). To address the postharvest darkening issue, investigations have been carried out mostly on two pinto bean cultivars- CDC Pintium [regular darkening (RD)] and 1533-15 [slow darkening (SD)] (Beninger et al., 2005; Junk-Knievel et al., 2008; Elsadr et al., 2011; Felicetti et al., 2012; Elsadr et al., 2015; Islam et al., 2020). Transcriptomic and metabolomic analysis performed in the seed coat tissues of CDC Pintium and 1533-15 identified several differentially expressed genes involved in PA biosynthesis and their transport, and accumulation of PA precursors (Duwadi et al., 2018). Furthermore, an allele of *P* gene, *p^sd^*, was found to cause SD phenotype in pinto beans (Islam et al., 2020).

**Figure 1:**
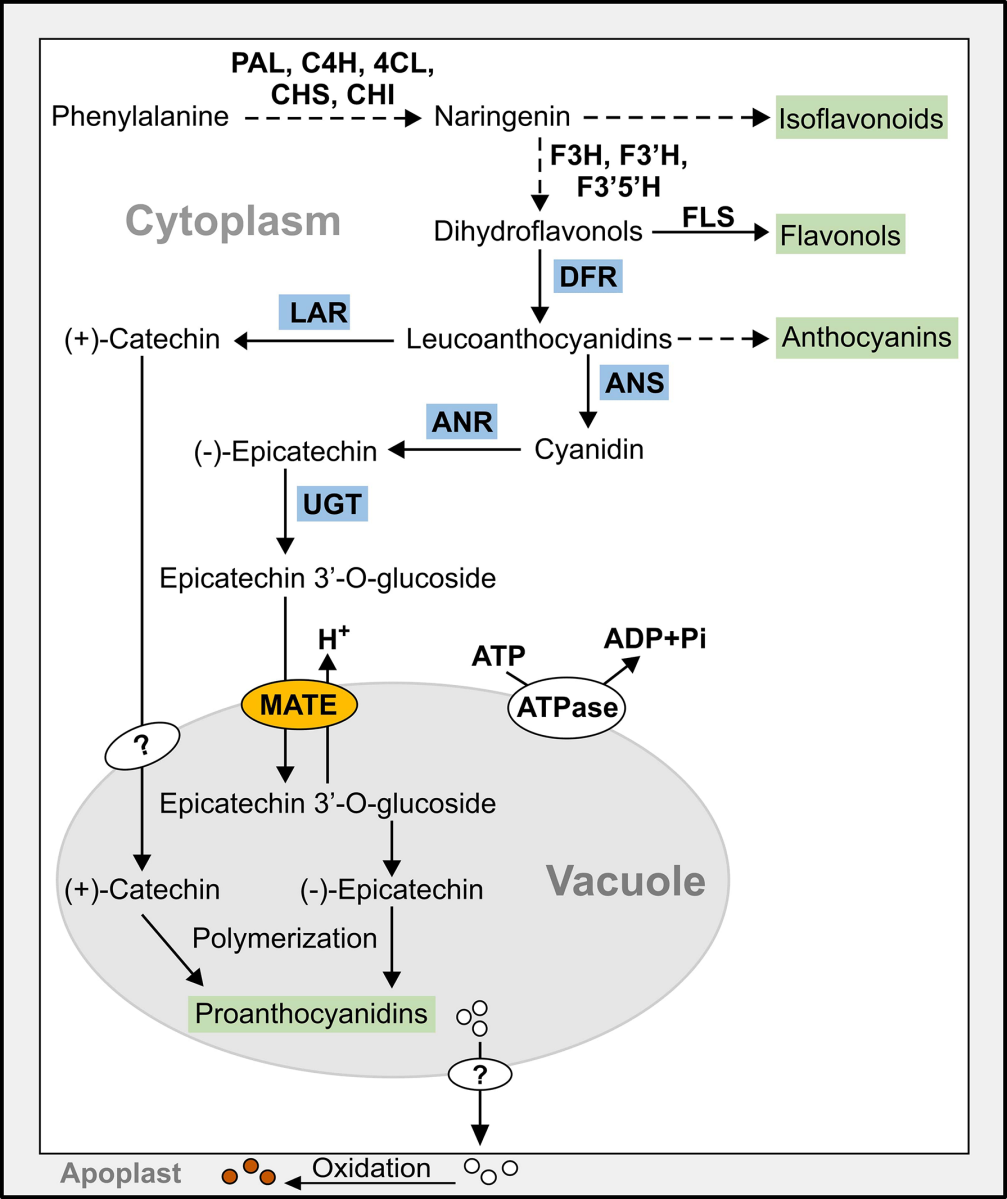
Simplified model of the flavonoid biosynthetic pathway leading to the synthesis of proanthocyanidins (PAs) in pinto bean. Dashed line indicates multiple steps, end products are highlighted in green, and late PA biosynthetic gene encoded enzymes are highlighted in blue. 4Cl, 4-coumarate-CoA ligase; ANR, anthocyanidin reductase; ANS, anthocyanidin synthase; C4H, cinnamate-4-hydroxylase; CHI, chalcone isomerase; CHS, chalcone synthase; DFR, dihydroflavonol reductase; F3’5’H, flavonoid 3′,5′-hydroxylase; F3’H, flavonoid 3′-hydroxylase; F3H, flavanone 3-hydroxylase; FLS, flavonol synthase; LAR, leucoanthocyanidin reductase; MATE, multidrug and toxic compound extrusion protein; PAL, phenylalanine ammonia lyase; UGT, UDP-glucosyltransferase.

PAs are oligomeric or polymeric flavonoids deposited in the vacuole of specific organs in plants (Dixon et al., 2005). The late flavonoid biosynthetic genes [*DIHYDROFLAVONOL 4-REDUCTASE (DFR), LEUCOANTHOCYANIDIN REDUCTASE (LAR), ANTHOCYANIDIN SYNTHASE (ANS)*, and *ANTHOCYANIDIN REDUCTASE (ANR)*] regulate the biosynthesis of (+)-catechin and (-)-epicatechin, the precursors of PA (Debeaujon et al., 2003; Pang et al., 2007) (**Figure 1**). The (+)-catechin and (-)-epicatechin are synthesized and glycosylated in the cytosol after which they are transported to the vacuole for polymerization (Marinova et al., 2007; Pang et al., 2007; Pang et al., 2008). Two vacuolar MATEs, TRANSPARENT TESTA12 (TT12) in Arabidopsis (Marinova et al., 2007) and MtMATE1 (Zhao and Dixon, 2009) are involved in the transport of PA monomers to the vacuole. Both AtTT12 and MtMATE1 could use only epicatechin 3’-O-glucoside (E3’G) as the substrate but not its aglycone.

Despite that plants contain large number of MATEs with diverse roles, only a few are functionally characterized. To date, no MATE is identified in common bean. Here we report that common bean genome contains 59 putative MATE transporters. The phylogenetic analysis of PvMATE candidates with functionally characterized MATEs from other plant species identified PvMATEs possibly involved in the transport of PA precursors. Finally, expression analysis of candidate PvMATEs in seed coat during development, their subcellular locations, genetic complementation and metabolite assays identified PvMATE8 as a transporter of PA monomers in pintos. Our results suggest the flow of PA monomers and feedback regulation of the upstream biosynthetic genes by the accumulation of E3’G in the cytosol.

## Materials and methods

### *In silico* analysis

For the identification of *PvMATE* genes, *P. vulgaris* whole genome sequence was accessed from Phytozome 13 (https://phytozome-next.jgi.doe.gov/info/Pvulgaris_v2_1), a Plant Comparative Genomics portal (Schmutz et al., 2014). The database was searched using the keyword “MATE”. Each of the obtained hits was used as a query sequence to perform BLAST search against the *A. thaliana* genome (Lamesch et al., 2011). The protein family information was confirmed with PANTHER (http://pantherdb.org/) and PFAM (https://pfam.xfam.org/). Theoretical isoelectric point (pI) and molecular weight (MW) of candidate PvMATE proteins were computed by ExPASy Compute pI/Mw (https://web.expasy.org/compute_pi/) (Wilkins et al., 1999). The subcellular localization of the PvMATE proteins was predicted using WoLF PSORT (http://www.genscript.com/wolf-psort.html) (Horton et al., 2007) and the numbers of transmembrane helices were predicted by TMHMM Server v2.0 (http://www.cbs.dtu.dk/services/TMHMM/) (Krogh et al., 2001). Amino acid sequences of candidate PvMATEs were used to find their homologs in *P. vulgaris* Ul111 v1.1 (https://phytozome-next.jgi.doe.gov/info/PvulgarisUI111_v1_1).

Location of *PvMATE*s and chromosome length (in Mbp) were obtained from Phytozome 13. The chromosomal locations of candidate *PvMATE* genes were illustrated by MapChart 2.32 (Voorrips, 2002).

### Phylogeny and protein motif analysis of candidate MATEs in *P. vulgaris*

For phylogenetic analysis, protein sequences of candidate MATEs from *P. vulgaris* and functionally characterized MATEs from other plant species were aligned in ClustalO (https://www.ebi.ac.uk/Tools/msa/clustalo/) and a tree was constructed by the neighbor-joining method with the *p*-distance statistical matrix and bootstrap set to 1000 using MEGAX software (Sievers et al., 2011; Kumar et al., 2018). The tree was visualized using iTOL (Letunic and Bork, 2021).

Conserved protein sequence motifs were identified using Multiple EM for Motif Elicitation (MEME) (http://meme-suite.org/). MEME identifies the occurrence of statistically significant motifs at specific positions of the given sets of sequences (e.g. with no gaps and lowest E- value) and ranks them (Bailey et al., 2009). For motif discovery on MEME, site distribution option was selected for zero or one occurrence (of a contributing motif site) per sequence, the maximum number of motifs was set at 12, and the remaining parameters were left as default classical mode. The motif composition of PvMATE proteins was visualized by TBtools (Chen et al., 2020a).

### Tissue-specific expression of candidate *PvMATE* genes

The tissue-specific expression analysis was performed for two different cultivars of common bean from publicly available data. For the common bean G19833, transcript abundance data were retrieved from Phytozome 13 in FPKM values (Schmutz et al., 2014). For the common bean cultivar Negro Jamapa, publically available RNAseq dataset (O'Rourke et al., 2014) was retrieved from the NCBI SRA database and processed and mapped on reference genome *P. vulgaris* v2.1 using CLC Genomic Work Bench (Release 20, www.qiagenbioinformatics.com/products/clc-genomicsworkbench/) with default settings permitting maximum two mismatches. The RPKM values were calculated from their normalized expression. Heatmaps were generated using log_2_-transformed normalized transcript abundances values using TBtools (Chen et al., 2020). Gene cluster in the newick tree was generated in MEGAX and imported into the heatmaps. RNAseq data for seed coat tissues were obtained from Duwadi et al. (2018).

### Plant materials and growth conditions

Wild type *A. thaliana* ecotype Wassilewskija (Ws-2) and *tt12* mutant seeds (Accession CS5740) were obtained from the Arabidopsis Biological Resource Center (Columbus, OH). Pinto bean cultivars CDC Pintium and 1533-15 were obtained from Dr. Kirstin Bett, University of Saskatchewan, Canada.

All plants were grown on Pro-Mix PGX soil in a growth room under 16 h light at 25°C and 8 h dark at 20°C cycle. Surface sterilized pinto bean seeds were grown under a light intensity of 300-400 μmol photons/m^2^/s. *A. thaliana* seeds were sterilized and grown on Murashige and Skoog medium for 1 week, transferred to soil and grown at a light intensity of 150 μmol photons/m^2^/s. *N. benthamiana* plants were grown under the light intensity of 80 μmol photons/m^2^/s.

### Gene cloning

The coding regions of candidate *PvMATE*s were amplified with gene-specific primers (**Supplementary Table S6**) and cloned into pDONR-Zeo (Invitrogen) using BP clonase (Invitrogen), followed by transformation into *Escherichia coli* DH5α *via* electroporation. The recombinant entry plasmids were verified by sequencing and recombined into two destination vectors separately, pEarleyGate101 and pEarleyGate100, using LR clonase (Invitrogen) for subcellular localization and plant transformation, respectively (Earley et al., 2006; Karimi et al., 2007). The recombinant destination plasmids were transformed into *Agrobacterium tumefaciens* GV3101.

For the cloning of *UGT72L1* (Medtr8g009063), the gene was synthesized and cloned into pUC57 (BioBasic) with *Bam*HI and *Sal*I restriction sites at 5’ and 3’ ends of the coding sequence, respectively and sub-cloned into pMALc2X vector (Addgene). The pMAL-UGT72L1 construct was transformed into *E. coli* NoveBlue DE3 cells (Novagen).

### RNA isolation and RT-qPCR analysis

Total RNA from bean seed coat was extracted using RNeasy Plant Mini kit (Qiagen). On-column DNA digestion was performed using DNase I (Promega). cDNA was synthesized from total RNA (1.0 μg) using the Platinum™ Quantitative RT-PCR ThermoScript™ One-Step System (Invitrogen). For qRT-PCR, cDNA from bean seed coat was used as template with SsoFast EvaGreen Supermix (BioRad) and gene-specific primers (**Supplementary Table S6**). The expression was normalized to *PvUBQ* (Accession *Phvul.007G052600.1*). Each experiment included two biological replicates, with three technical replicates for each biological replicate. The data were analyzed using CFX Maestro (Bio-Rad).

### Subcellular localization and confocal microscopy

For subcellular localization, *A. tumefaciens* harboring pEarleyGate101-PvMATE from pinto bean cultivar CDC Pintium was infiltrated into leaves of 4-6 week-old *N. benthamiana* plants using the method described in Sparkes et al. (2006). The epidermal cell layers of the infiltrated leaves were visualized 48 h post-infiltration using an Olympus FV1000 confocal microscope (Olympus Corporation) with a 60× water immersion objective lens. To confirm the localization, *A. tumefaciens* containing vacuole (VAC-ck CS16256) and ER (ER-ck CS16250) markers translationally fused with Cyan Fluorescence Protein (CFP) were co-infiltrated with the PvMATE constructs in 1:1 ratio (Nelson et al., 2007). Fluorescence for CFP and Yellow Fluorescence Protein (YFP) were visualized at the excitation wavelength of 514 nm and 434 nm, and emission of 530-560 nm and 470-500 nm, respectively. To visualize the co-localization of the YFP and CFP signals, the ‘Sequential Scan’ tool was used.

### Complementation assay and seed coat staining

Arabidopsis *tt12* plants were transformed with *A. tumefaciens* harboring pEarleyGate100-PvMATE using the floral dip infiltration method (Clough and Bent, 1998). T1 plants were selected on plates containing Murashige and Skoog media supplemented with phosphinothricin (7.5 mg/mL). The phosphinothricin-resistant seedlings were transferred to soil, followed by genotyping to confirm the presence of respective transgenes.

To confirm PA accumulation in transgenic seeds, seeds were stained with 4-dimethylamino-cinnamaldehyde (DMACA) following the method described by Li et al. (1996). Briefly, seeds were stained with 0.5% (w/v) DMACA for 36 h in dark followed by de-staining with 70% ethanol. Photographs were taken using an SMZ1500 dissecting microscope with camera (Nikon).

### Synthesis of E3’G

#### Expression and purification of UGT72L1

MBP-UGT was expressed and purified according to the New England Biolabs “pMAL Protein Fusion and Purification System” instruction manual. A single colony of *E. coli* NovaBlue (DE3) harboring pMAL-UGT was inoculated into 5 mL Rich medium containing 100 µg/mL ampicillin and 12.5 µg/mL tetracycline, and cells were grown overnight at 37°C. The next day, the overnight culture was transferred to 500 mL Rich medium. The cells were grown to OD_600_ of 0.6 at 37°C, IPTG was added to a final concentration of 0.3 mM. The culture was incubated for 16 h at room temperature. Cells were harvested by centrifugation at 4,500 × *g* at 4°C for 10 min. Pellet was re-suspended in 15 mL of column buffer (20 mM Tris-HCl pH 7.5, 200 mM NaCl, 1 mM EDTA) containing 1 mg/mL lysozyme and cells incubated on ice for 30 min.

Cells were lysed by French Press, sonicated 5× for 30 sec and spun for 30 min at 4°C at 25,000× *g*. Four mL of Amylose resin (New England Biolabs) were added to Poly-Prep<sp>® chromatography column (Bio-Rad) and washed three times with column buffer. The sample was added to the column and incubated rotating at 4°C for 30 min. The column was washed 10 times with column buffer, and 8 mL of elution buffer (20 mM Tris-HCl pH 7.5, 200 mM NaCl, 1mM EDTA, 20 mM maltose) were added to a capped chromatography column and incubated at 4°C for 30 min. The column was uncapped and the flow-through collected and concentrated on an Amicon Ultra-15 centrifugal filter unit (Millipore Sigma) to 5 mL. Samples were desalted (2.5 mL each) on PD-10 columns (Cytiva) into 3.5 mL of 100 mM Tris-HCl pH 7.5.

### HPLC analysis and semi-preparative HPLC isolation of E3’G

MBP-UGT was assayed in a reaction volume of 100 μL containing 10 mM Tris-HCl pH7.5, 10 μg protein, 0.1 mM epicatechin, and 0.2 mM UDP-Glucose. All assays were performed in triplicate for 3 h at 30°C followed by 21 h at room temperature. Samples were deproteinized on Nanosep Centrifugal Devices Omega™ Membrane - 3K (Pall) and analyzed by HPLC. The epicatechin standard and the E3’G product were analyzed under the following conditions on an Agilent 1260 system [binary pump (G7112B), auto-sampler (G7129A), column compartment (G1316A), diode array detector (G4212B)] (**Supplementary Figure S1**). A 10 µL injection was made onto an Agilent Poroshell EC-C18, 4.6 mm × 100 mm × 2.7 µm column. The flow rate was 1mL/min with a binary solvent system comprised of solvent A: H_2_O + 0.1% TFA; B: Acetonitrile (MeCN) + 0.1% TFA. The column was maintained at 32^o^C and peaks were monitored at 210 nm. The gradient consisted of 98% A for 1 min, followed by a linear increase to 30% B at 16 min, 100% B at 16.5 min, 100% B at 18 min, a return to 98 % A at 18.5 min, and re-equilibration at 98% A until 20 min.

E3’G was isolated on the same system using a fraction collector (G1364C). The epicatechin standard and glycosylated product were injected onto a Phenomenex Gemini<sp>® 10 mm × 150 mm × 5 µm (110Å) column at 4 mL/min under the same gradient conditions as the analytical column method. Collected fractions were pooled and dried using a BUCHI Rotovapor<sp>®.

### Metabolite extraction and LC-MS analysis

Arabidopsis seeds (0.03-0.04 g) were ground with liquid nitrogen and metabolites were extracted in methanol:water (4:1, v/v). Samples were sonicated for 15 min followed by centrifugation at 11,000 × *g* for 10 min at 4°C. The supernatant was filtered through a 0.22 mm PTFE syringe filter (Chromatographic Specialties, Canada), and then dried under nitrogen gas. The dried metabolite extracts were dissolved in methanol:water (1:1, v/v) and used in LC-MS analysis.

LC-MS analysis was performed as described in Anguraj Vadivel et al. (2019), where metabolites were extracted from all seed samples and analyzed under the same condition. The raw chromatogram data for (HESI-) were converted to “mzxml” using ProteoWizard (Kessner et al., 2008) and then processed to obtain peak areas using mzMine (Pluskal et al., 2010). Metabolites were identified by comparing the chromatographic behavior with commercial standards for those which were available and synthesized E3’G (**Supplementary Table S7**; **Supplementary Figure S1**).

## Results and discussion

### Common bean genome contains 59 *MATE* genes

**Figure 2:**
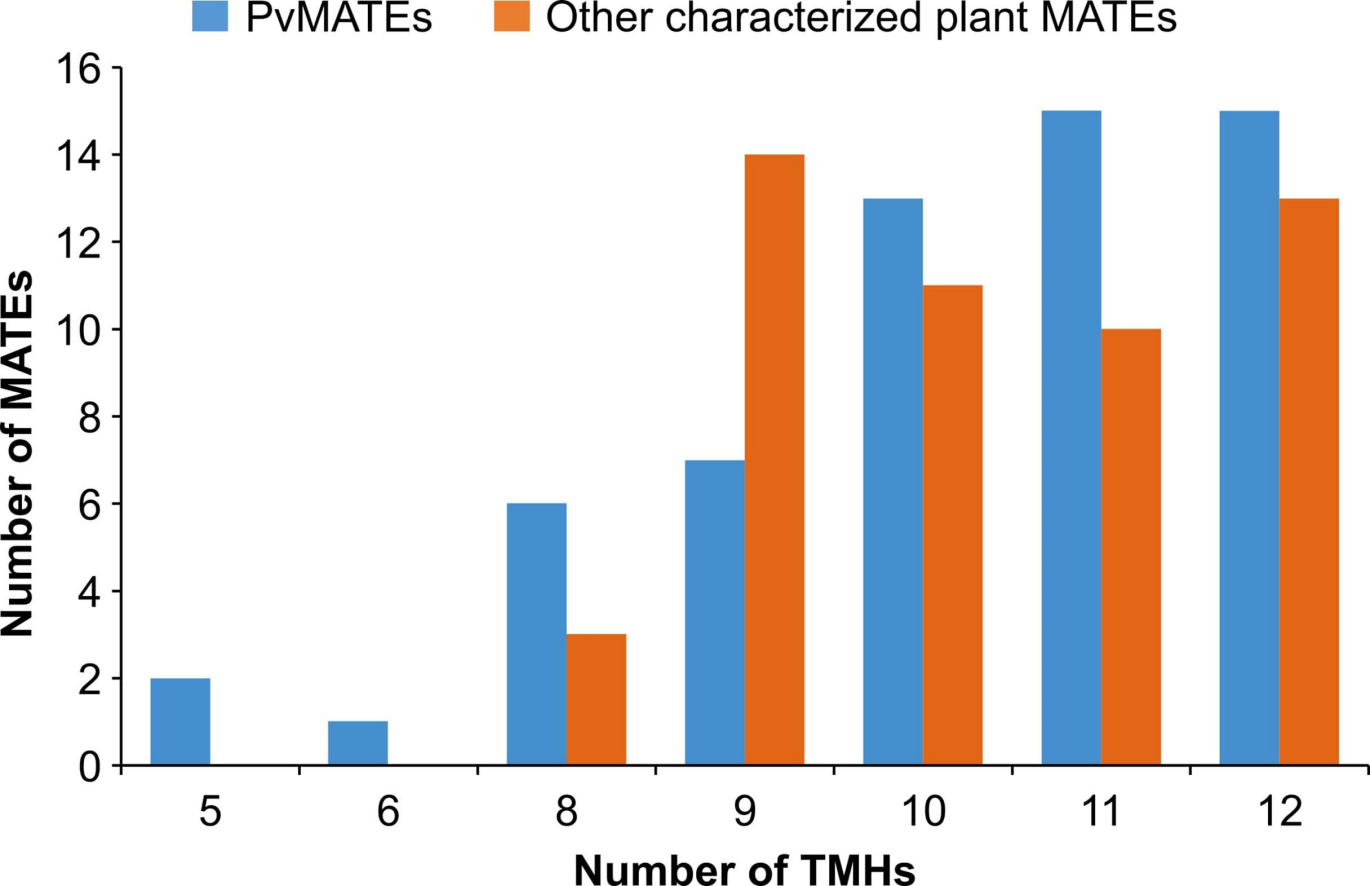
Number of predicted transmembrane helices in 59 candidate PvMATEs and 51 other characterized plant MATEs. Transmembrane helices predictions were performed using TMHMM v 2.0.

To identify the *MATE* genes in common bean, we searched the whole genome sequence of common bean G19833 available at Phytozome 13 (Schmutz et al., 2014) using the keyword ‘MATE’. From a total of 61 hits, 2 were not MATEs as they neither contained any “MatE” motif nor their sequences matched with any Arabidopsis and common bean MATEs. After excluding those 2, a total of 59 *PvMATE* genes were identified in common bean genome. MatE motifs share highly conserved amino acid sequences with other proteins that also contain transmembrane helices and have transport functions (Putman et al., 2000). Additional BLAST searches using the protein sequence of each of the 59 candidate *PvMATE* as the query against the common bean G19833 genome did not identify any additional candidate confirming that no *PvMATE* candidate was missed in the search. The calculated molecular masses of candidate PvMATEs ranged from 43 to 60.4 kDa, except for the Phvul.004G152301.1 (24.6 kDa) and Phvul.004G152601.1 (23 kDa). Of the 59 PvMATEs, 15 contain 12 transmembrane helices. As shown in **Figure 2**, 43 PvMATEs contain transmembrane helices ranging from 10-12 while others have fewer of them. Plant MATE proteins usually contain 12 transmembrane helices (Saier and Paulsen, 2001; Tanaka et al., 2013; Takanashi et al., 2014). Analysis of the transmembrane helices in 51 plant MATEs functionally characterized until now (**Supplementary Table S1**) revealed the presence of 8 to 12 transmembrane helices in them (**Figure 2**), suggesting that a minimum of 8 transmembrane helices are required for MATE proteins to be functional in plants and that the candidate PvMATEs with less than 8 transmembrane helices are possibly non-functional. Since the subcellular localization of a protein can shed light on its function, biosynthetic pathway determination and substrate identification, we predicted the subcellular localization of the candidate PvMATEs. The results indicated that the candidate PvMATEs were mostly localized on the plasma membrane (74.57%), cytoplasm (8.47%), chloroplast (8.47%) and vacuole (6.7%). Detailed characteristics of PvMATEs are shown in **Supplementary Table S2**.

Recently, common bean cultivar pinto UI111 genome sequence has been released (https://phytozome-next.jgi.doe.gov/info/PvulgarisUI111_v1_1). Candidate PvMATEs from common bean G19833 share 98 to 100% amino acid sequence identity with cultivar pinto UI111 (**Supplementary Table S3**). The protein sequence alignments of the two candidate PvMATEs with shorter peptides- Phvul.004G152301 and Phvul.004G152601, matched with the C- terminal halves of PvUI111.04G149500 (97% identity) and PvUI111.04G149700 (100% identity) (**Supplementary Figure S2**), indicating a possible discrepancy in the sequence assembly.

**Figure 3:**
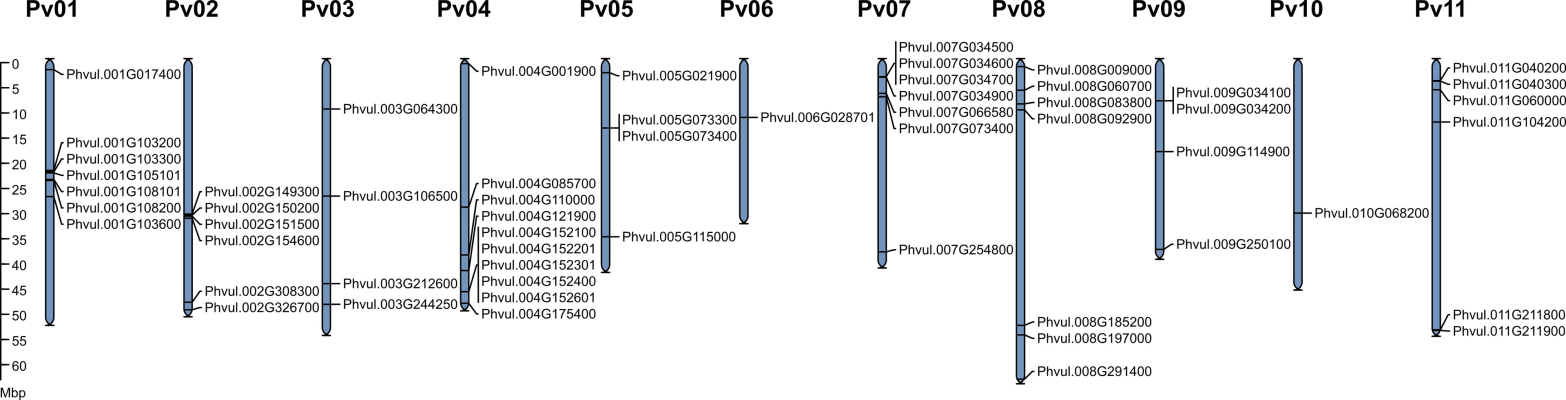
Genomic distribution of *PvMATE* genes in *P. vulgaris* landrace G19833 chromosomes. The chromosome numbers are indicated above each chromosome and drawn to a scale in megabase pairs (Mbp). The chromosome size is indicated by its relative length using the information from Phytozome 13.

Except for *Phvul.L001777* scaffold_477 and *Phvul.L008943* scaffold_15, *PvMATE* genes are randomly distributed on all 11 chromosomes of common bean (**Figure 3**). On chromosome 1, *Phvul.001G103300* and *Phvul.001G105101* share 99.79% (1 SNP) while *Phvul.001G103600* and *Phvul.001G108101* share 99.53% (6 SNPs) sequence identity at the nucleotide level. These paralog pairs possess 100% sequence identity in their protein sequences. When a blast search was performed with the protein sequence of these paralogs against *P. vulgaris* UI111 genome, Phvul.001G103300 and Phvul.001G105101 pair identified only one homolog PvUI111.01G101100 (99% identity), and Phvul.001G103600 and Phvul.001G108101 identified PvUI111.01G104500 (100% identity) (**Supplementary Table S3**). Additionally, the protein sequence of Phvul.007G034600 shares 100% and 86% sequence identity with PvUI111.07G034000 and PvUI111.07G034100, respectively. Finally, PvUI111.01G112600 in pinto UI111 has a shorter peptide (143 amino acids) which also has 99% sequence identity with Phvul.001G103600 and Phvul.001G108101 paralog pair. Thus the total number of candidate *MATEs* in pinto UI111 is 58. From an evolutionary perspective, *P. vulgaris* landrace G19833 is of Andean origin (Race Peru) (Schmutz et al., 2014) and cultivar pinto UI111 is of middle American origin (Race Durango) (Singh et al., 1991). The variation in the total number of *MATE* genes in these two cultivars is possibly due to gene duplication on chromosome 1, and the observed polymorphisms in the gene sequences between cultivars are more likely due to their different geographic origins (Chacón S et al., 2005; Wang et al., 2015).

To determine the chromosomal distribution of *MATE* genes and their genome organization in *P. vulgaris* G19833 and pinto UI111, chromosome maps were constructed (**Figures 3**, **S3**). For G19833, as shown in **Figure 3**, the gene density per chromosome is uneven. Except for *Phvul.L001777* scaffold_477 and *Phvul.L008943* scaffold_15, *PvMATE* genes are distributed on all 11 chromosomes. The majority of *PvMATE*s on chromosomes 1 and 2 are located near the center, whereas others are located at the chromosomal ends. Most of the chromosomes contain multiple *PvMATE* genes clustered together except for chromosomes 6 and 10 that contain a single candidate *PvMATE*. The same pattern was observed for pinto UI111 (**Supplementary Figure S3**; **Supplementary Table S4**). Clusters of the members of the same gene family indicate consecutive gene duplication events resulting from unequal crossing over from the sister chromatids during mitosis or homologous chromosomes during meiosis (Moore and Purugganan, 2003; Reams and Roth, 2015). This duplication event is often accelerated if repeat sequences flank the genic regions. Thus, it is common to observe gene family members in a tandem arrangement in the same region of a chromosome.

### Candidate PvMATEs are distributed into four clades

**Figure 4:**
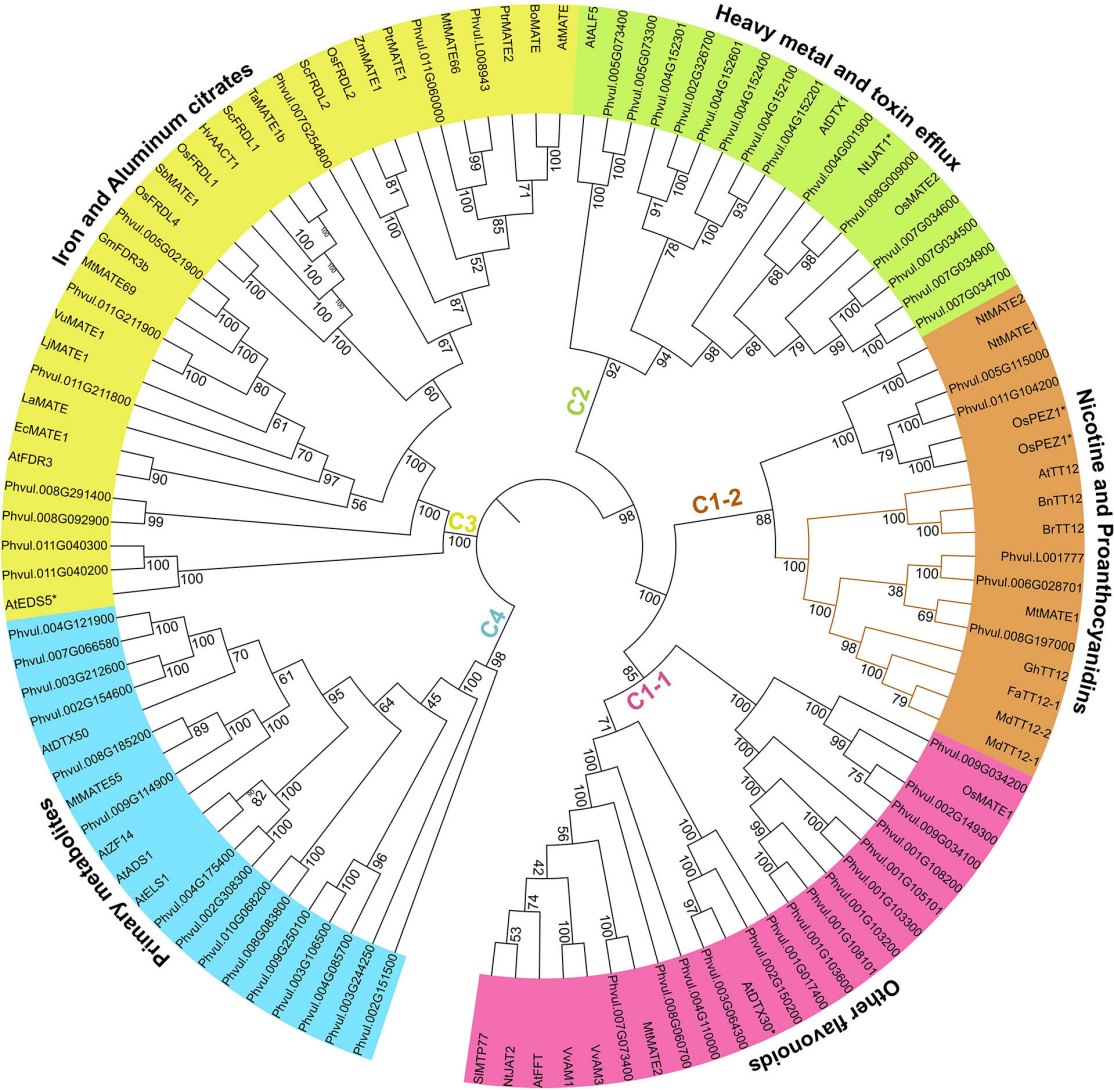
Phylogeny analysis of 59 PvMATEs from *P. vulgaris* G19833 and 51 characterized MATEs from other plant species. The tree was constructed in MEGAX using the neighbor-joining method. The bootstrap values are shown in percentage (1000 replicates) on the tree nodes. Different clades and sub-clades are highlighted using different colors and clade-specific substrates specifications are shown on the arcs outside the tree. Based on the literature, any characterized MATEs with functional exceptions are shown with asterisks (*) with the name.

Membrane transporters are a diverse group of proteins that differ in structure, energy coupling mechanism, and substrate specificity (Lee et al., 2008). Among them, MATE proteins are highly specific toward their substrates. They are reported to transport a wide range of small molecules that include metal ions to complex primary and specialized metabolites (Takanashi et al., 2014). To date, there are 51 functionally characterized MATEs from diverse plant species that have been demonstrated to play roles in transporting organic acids (e.g., citrates) and metal ions (Fe3+, Al3+, Cd2+, etc.), flavonoids (e.g., anthocyanin, PAs), and alkaloids (nicotine) (**Supplementary Table S1**). A phylogenetic analysis of the 59 candidate PvMATEs along with the 51 functionally characterized plant MATE proteins categorized PvMATEs into four primary clades (C1 to C4) with the transport potential of distinct substrate types (**Figure 4**). MATE proteins from *M. truncatula* (Min et al., 2019), *Cajanus cajan* (Dong et al., 2019), *Camellia sinensis* (Chen et al., 2020b), *Solanum lycopersicum* (Santos et al., 2017), *Gossypium hirsutum* (Xu et al., 2019) and soybean (Liu et al., 2016) also showed similar groupings.

C1 clade is the most extensively studied clade that contains 20 functionally characterized MATE proteins from diverse plant species. MATEs grouped in C1 clade transport flavonoids or alkaloids into the vacuole and is further subdivided into C1-1 and C1-2. C1-1 sub-clade contains 15 candidate PvMATEs clustered with 8 previously characterized plant MATEs. Anthocyanin transporters of C1-1 sub-clade includes MtMATE2 for malonylated flavonoid glucosides transport in *M. truncatula* (Zhao et al., 2011), SlMTP77 for anthocyanin transport in tomato (Mathews et al., 2003), AnthoMATEs, VvAM1 and VvAM3 with overlapping transport functions of acylated anthocyanins in *Vitis vinifera* (Gomez et al., 2009), and AtFFT for glycosylated flavonols (kaempferol 3,7-O-diglucoside) transport, which are the precursors of anthocyanin or PA monomers (Thompson et al., 2010). MATEs reported in C1-1 are ATP-driven and mostly expressed in floral organs and leaves.

A total of 5 PvMATEs grouped with 12 characterized plant MATEs in the sub-clade C1-2 that contains two distinct branches for nicotine and PA transport. The PA transporter groups of MATEs include TT12 from multiple plant species that are involved in the transport of glucosides of epicatechin (E3’G) and cyanidins (Cy3’G) (Marinova et al., 2007; Chai et al., 2009; Shoji et al., 2009; Zhao and Dixon, 2009). In the vacuole, the E3’G gets hydrolyzed releasing the key monomer of PA that produces brown pigment in the apoplast. Deficiency in the transport of E3’G by MATE to the vacuole will result into the alteration of PA accumulation producing light yellow seed coat color in Arabidopsis *tt12* mutants. All TT12 orthologs are Mg^2+^-ATP flavonoid/H^+^-antiporter (Marinova et al., 2007; Zhao and Dixon, 2009; Gao et al., 2016). Characterized MATEs of this subgroup do not transport catechins, another major monomer of PA, indicating the presence of a different route of catechin transport (Zhao and Dixon, 2009). Additionally, it has been reported that C1 clade MATEs are regulated by a MYB transcription factor and a cytosolic enzyme is required for the modification of flavonoids before they can be transported by their respective MATEs (Mathews et al., 2003; Zhao and Dixon, 2009). Three PvMATEs, Phvul.008G197000, Phvul.006G028701 and Phvul.L001777 cluster together with a legume PA transporter MtMATE1 suggesting their similar role.

Clade C2 contains 14 PvMATEs. To date, only 4 MATEs belonging to C2 clade have been functionally characterized participating in the transport of several heavy metals and complex toxic molecules. Arabidopsis Aberrant Lateral-root Formation 5 (ALF5/DTX19) and DTX1 have roles in the detoxification of heavy metals, alkaloids and antibiotics (Diener et al., 2001; Li et al., 2002). Tobacco Jasmonate-inducible Alkaloid Transporter 1 (NtJAT1) is known for vacuolar sequestration of alkaloids like nicotine, anabasin, hyoscyamine, and berberine (Morita et al., 2009). OsMATE2 was identified with diverse functions when expressed in Arabidopsis including, arsenic and pathogen response, flavonoid transport along with its homologue OsMATE1, which belongs to the clade C1-1 (Tiwari et al., 2014).

Ten candidate PvMATEs clustered in C3 clade that contained 12 functionally characterized metal ions and small molecule transporters from other plants. C3 clade MATEs are the transporters of metal ions and small molecules and one of the most studied group of MATEs in plants. Arabidopsis Enhanced Disease Susceptibility 5 (AtEDS5) plays a role as SA accumulator and provides pathogen and abiotic resistance (Serrano et al., 2013). AtEDS5 is the exception in C3 clade as other MATEs belonging to this group are involved in the transport of metal citrates. For example, Arabidopsis Ferric Reductase Defective 3 (FRD3) (Pineau et al., 2012), white/field lupin (*Lupinus albus*) LaMATE (Uhde-Stone et al., 2005), and MtMATE69 and MtMATE66 (Wang et al., 2017) can transport a range of di- or trivalent metal ions. The remaining members of this clade include *Sorghum bicolor* MATE1 (SbMATE1) (Magalhaes et al., 2007), *Oryza sativa* FRD3 like 1 and 4 (OsFRDL1 and OsFRDL4) (Yokosho et al., 2009; Yokosho et al., 2011), *Hordeum vulgare* aluminum-activated citrate transporter 1 (HvAACT1) (Zhou et al., 2013), *Zea mays* MATE1 (ZmMATE1) (Maron et al., 2013), AtMATE/AtDTX42 (Liu et al., 2009), *Brassica oleracea* MATE (BoMATE) (Wu et al., 2014) are Al^3+^ transporters and some are often reported to translocate Fe^3+^.

C4 clade contained 15 candidate PvMATEs grouped with 5 MATEs from Arabidopsis and *M. truncatula* that are involved in the transport of primary metabolites such as hormones including abscisic acid (ABA), salicylic acid (SA), auxin or iron homeostasis. The characterized MATE hormon transporter are Activated Disease Susceptibility 1 (AtADS1) (Sun et al., 2011), Arabidopsis Detoxification efflux carrier 50 (AtDTX50) (Zhang et al., 2014) and AtZF14, also known as Bush and Chlorotic Dwarf 1 or Abnormal Shoot 4 are mitochondrial proteins (Burko et al., 2011) in *A. thaliana* and MATE55 in *M. truncatula* (Wang et al., 2017).

Despite the fact that most plant MATEs are substrate-specific, some MATE proteins are able to transport multiple types of substrates. For example, OsMATE1 transports mainly toxic metals such as arsenic, however, it also functions as glutathione-S-transferase for anthocyanin (Tiwari et al., 2014). OsMATE1 clusters with the anthocyanin transporters in the sub-clade C1-1 (**Figure 4**), suggesting it may also have role in anthocyanin transport. Similarly, AtDTX30, OsPEZ1 and OsPEZ2 are grouped in the clade C1, even though they have not yet been demonstrated to transport flavonoids. AtDTX30 regulates auxin synthesis with aluminum transport (Upadhyay et al., 2020) while OsPEZ1 and OsPEZ2 are reported to transport iron (Bashir et al., 2011; Ishimaru et al., 2011). Regardless of some discrepancies observed, the grouping of PvMATEs into specific clades and elucidation of their phylogenetic relationships with functionally characterized plant MATE transporters suggest their similar function and substrate specificity.

**Figure 5:**
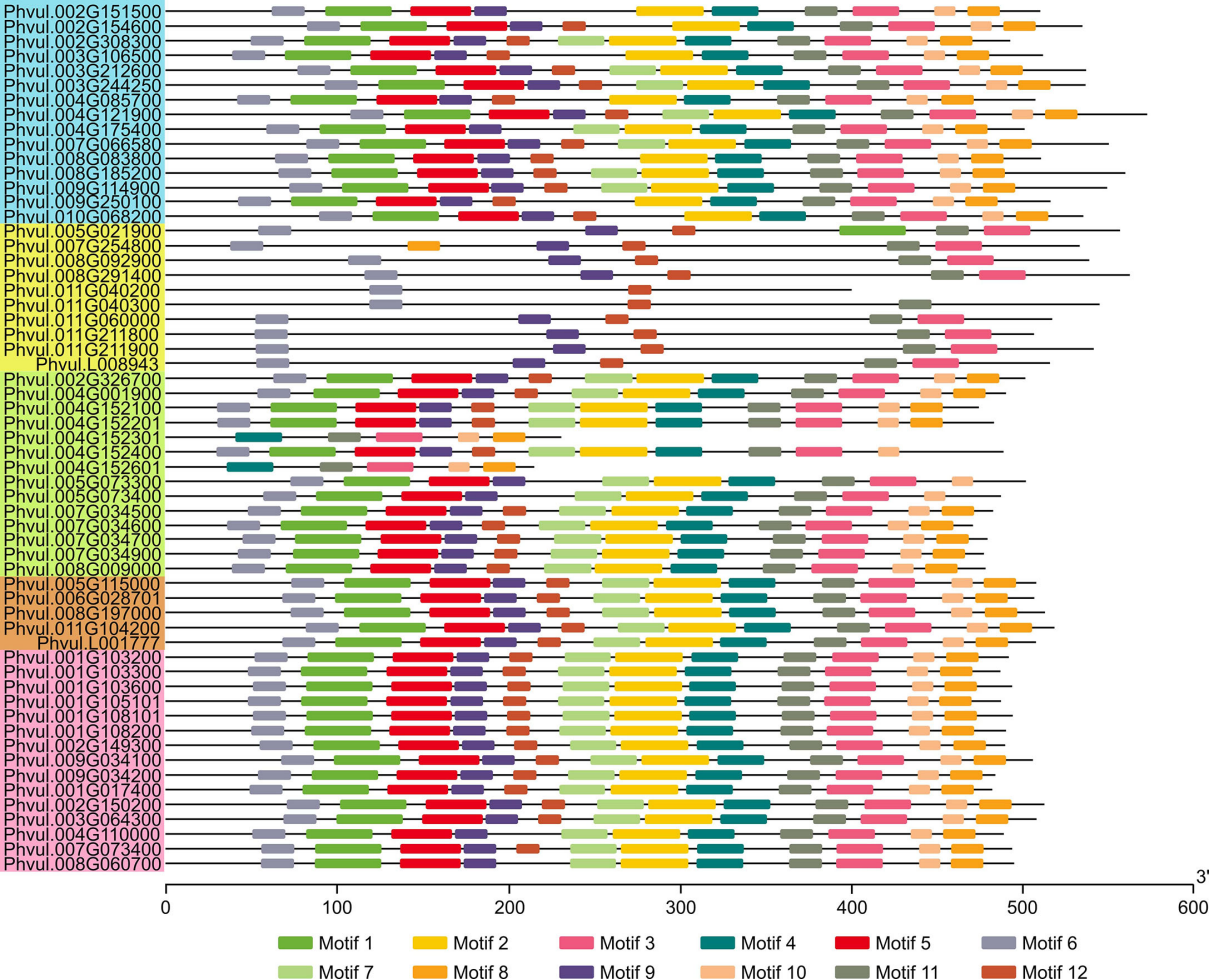
Conserved protein motifs of the MATE family in common bean. Colored boxes on lines are the conserved motifs and for protein length estimation, scale is provided on the bottom in amino acids units. The logo presentation of the conserved sequence is given in **Supplementary Figure S4**. PvMATEs are listed according to the order of clades and sub-clades from C1 to C4 as the phylogenetic tree in **Figure 4** and accordingly color-coded.

A motif search in the candidate PvMATEs revealed that they contain conserved sequence within each clade. Since plant MATEs usually contain a maximum of 12 transmembrane helices and those regions are relatively conserved than the rest of the sequences (Takanashi et al., 2014), for the motif search we used the input request for the number of motifs as 12. The results revealed that the majority of candidate PvMATEs irrespective of their clades contained most of the motifs (**Figures 5**, **S4**). A tightly conserved motif pattern was observed in the clade C1 that comprises the transporters of specialized metabolites. The exception was found in Phvul.004G110000 and Phvul.008G060700 from C1-1, which lacked the motif 12. Half of the members of C2 clade contained a similar motif pattern as of C1, while the rest half were variable. Phvul.004G152301 (24.66 kD) and Phvul.004G152601 (23.02 kD) are the two smallest MATE proteins in common bean, belong to C2 clade and contain only 5 motifs. The number of motifs in C4 clade varied between 10 to 12. Despite that the average molecular masses of PvMATEs belonging to C3 clade were the same as the PvMATEs in other clades, they contained only 5 to 6 motifs.

### Expression analysis of *MATE*s in *P. vulgaris*

**Figure 6:**
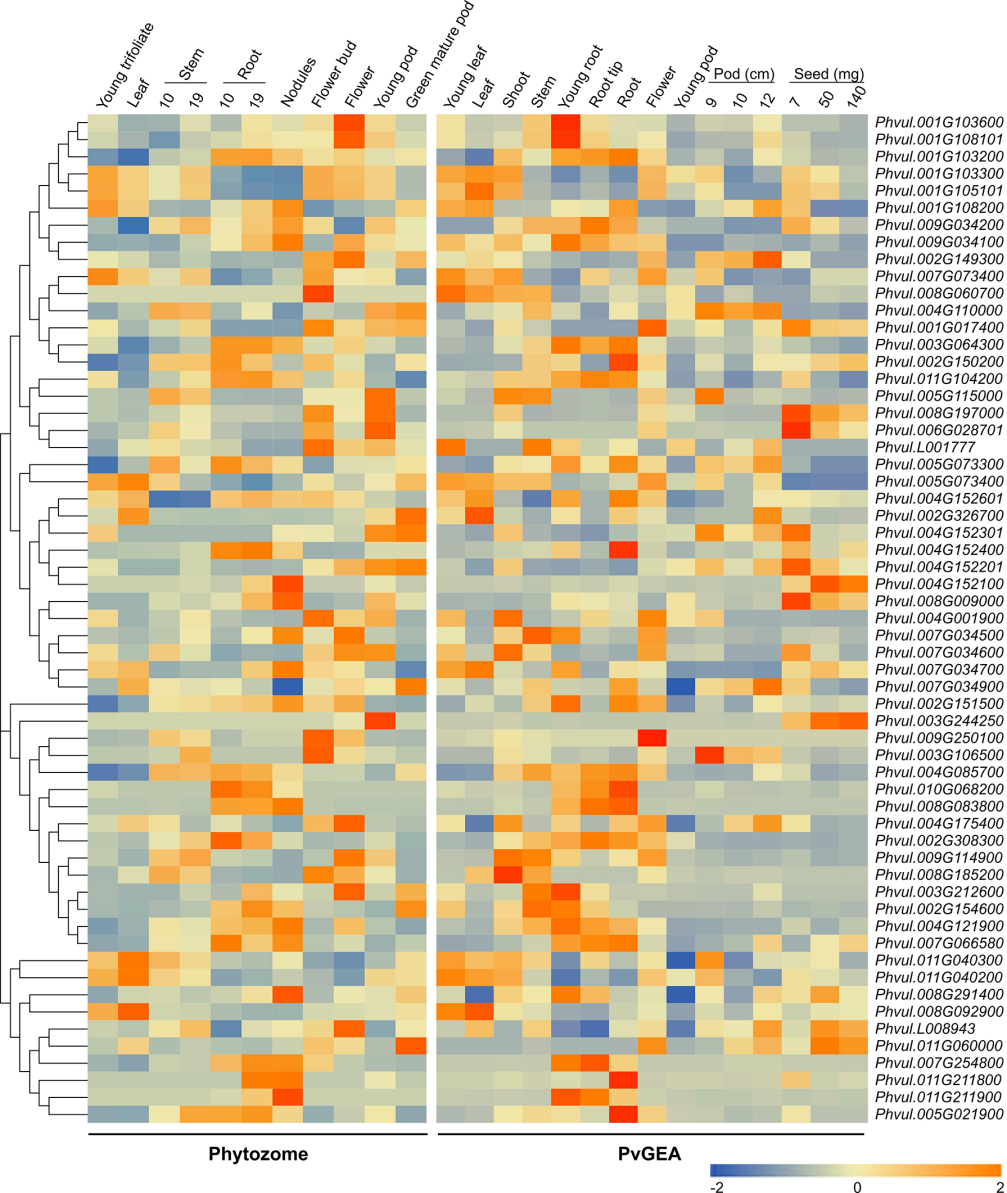
Heatmaps showing tissue-specific expression of *PvMATEs*. Phytozome for landrace G19833 and PvGEA for cultivar Negro Jamapa were used for data sources. The color key represents the relative transcript abundance in a row analyzed in different tissues with the log_2_ transformed values. Tissues with their developmental stages are labeled overhead. The gene cluster was imported from neighbor-joining-tree generated with PvMATE protein sequences in MEGAX.

To determine the tissue-specific expression of the *PvMATEs*, we retrieved the expression data from two publicly available datasets Phytozome 13 and PvGEA for common bean G19833 and Negro Jamapa, respectively. Common bean G19833 is a RD type brown color bean with large seed size while Negro Jamapa is a black bean with medium sized seed. The expression values from Phytozome 13 was from unpublished pipeline. This dataset consisted of the transcript abundance in tissues such as young trifoliate leaves, leaves, young pods, green mature pods, flower buds, flowers, nodules and stem and root at different growth stages. *PvMATE* expression in Fragments Per Kilobase of transcript per Million (FPKM) mapped reads ranged from 0.009341 (*Phvul.003G106500*, green mature pods) to 303.62 (*Phvul.011G104200*, root 19 days old plant) (**Figure 6**; **Supplementary Table S5**). The second dataset was obtained from the NCBI bioproject PRJNA210619, where the expression profiling was performed on common bean cultivar Negro Jamapa (O'Rourke et al., 2014). The experiment was conducted to study the gene expression upon nitrogen treatment in 7 different tissues (roots, nodules, stems, flowers, leaves, pods, and seeds) during the development. For our gene expression analysis, 15 untreated samples (controls) were selected to cover 7 distinct tissues and avoided the impact of nitrogen treatments (**Supplementary Table S5**). In Reads Per Kilobase of transcript per Million (RPKM) mapped reads values, *PvMATEs* expression varied from 0.0165 (*Phvul.011G060000*, shoot tip) to 406.78 (*Phvul.011G104200*, root tip). Heatmaps were generated to visualize the expression patterns of candidate *PvMATE* genes from the two data sources (**Figure 6**). A variation was observed between the RNAseq datasets which could be due to the difference in cultivars and/or growth stages (**Figure 6**). However, a common pattern of gene expression was observed for most of the candidate *PvMATEs* in the two datasets. For instance, *Phvul.006G028701* and *Phvul.008G197000* are both annotated as *Transparent testa 12 (TT12)* in the Phytozome 13 genome database that is involved in transport of specialized metabolites in Arabidopsis seeds (Marinova et al., 2007). These two genes show the highest expression in the seed tissues from PvGEA data and in the flower tissues from Phytozome 13, where seed expression data is not available.

The major function of MATE transporters is to detoxify and maintain homeostasis within the plant and with the environment (Eckardt, 2001). Root is the major route for several metal ion and xenobiotic effluxes, relating the highest expression in root. *PvMATE*s of clade 3 and 4 are speculated to be involved in metal ion and hormone transport. The higher accumulation of these *PvMATEs* in root, stem and flower supports their proposed functions. The expression of *PvMATEs* is strictly tissue and developmental stage-specific. For example, *PvMATE*s that express in the leaves rarely express in the roots and seeds, flowers and pods show similar expression pattern indicating developmental gene regulation.

### Potential PA transporter: *PvMATE*s differentially expressed in pinto bean RD and SD cultivars and localized in tonoplast

**Figure 7:**
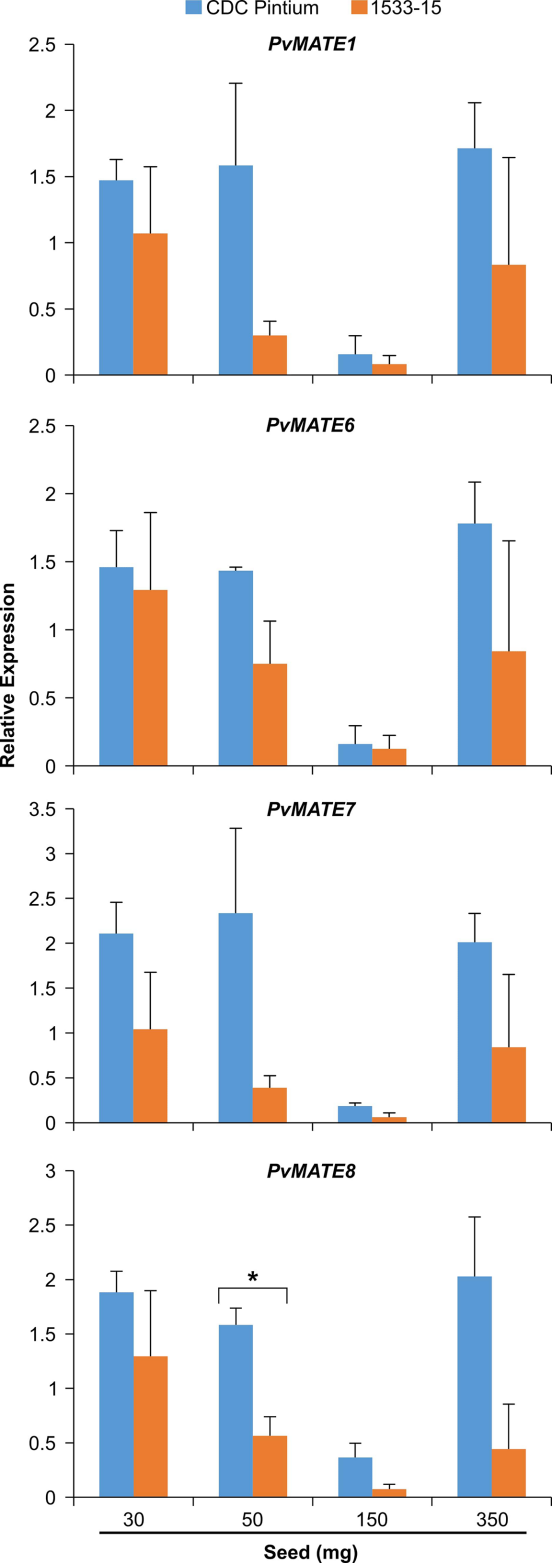
Differential expression of four selected *PvMATE* candidates for PA-specific function. Total RNA extracted from four different stages (30, 50, 150, and 350 mg seed weight) seed coat, collected from CDC Pintium (RD) and 1533–15 (SD) cultivars and qRT-PCR analysis was performed using gene-specific primers. Relative expression corresponds to the mean value in two biological replicates and three technical replicates per biological replicate. Error bar indicates SEM. Asterisk (*) indicates significant difference between the samples as determined by Student’s *t*-test.

Previously, we performed a transcriptome analysis to investigate the differential gene expression in the seed coats of two pinto bean cultivars, CDC Pintium and 1533-15, that differed in their postharvest seed coat color (Duwadi et al., 2018). Here, we searched the same dataset for the expression of *PvMATE*s and identified that out of 59 *PvMATEs*, 52 are expressed in the seed coat tissue. Among 52, 4 *PvMATE* genes showed differential expression (2 fold or higher) between CDC Pintium and 1533-15. Among the differentially expressed *PvMATE* genes, *Phvul.006G028701.1* (*PvMATE6*), *Phvul.007G034700.1* (*PvMATE7*) and *Phvul.008G197000.1* (*PvMATE8*) showed 3.1, 55.8 and 4.7 times higher expression, respectively in CDC Pintium compared to 1533-15 seed coat (**Table 1**). Although *PvMATE7* showed the highest fold change in expression, it had a low read count in 1533-15 (10.902) compared to *PvMATE8* (**Table 1**). On the other hand, *Phvul.001G103300.1* (*PvMATE1*) was expressed two-fold higher in 1533-15 than CDC Pintium seed coat. Among these 4 *PvMATE*s, only *PvMATE6* (*Phvul.006G028701.1*) and *PvMATE8 (Phvul.008G197000.1)* belong to the clade C1-2 (Nicotine and PA transporters). *PvMATE6* and *PvMATE8* transcripts have also been reported to accumulate at higher levels in darkening cranberry bean recombinant inbred lines compared to non-darkening cranberry beans (Freixas et al., 2017). *PvMATE1* belongs to the clade C1-1 (Anthocyanin transporters) and *PvMATE7* belongs to the clade 3 (toxin and heavy metal efflux) (**Figure 4**). The RNAseq was performed on a specific seed coat developmental stage (150 mg) (Duwadi et al., 2018), whereas expression of PA-specific genes start at the flowering and continues to seed maturation (Xu et al., 2015). Thus an additional expression analysis was performed for *PvMATE1*, *PvMATE6*, *PvMATE7* and *PvMATE8* using 4 different seed coat stages to avoid any discrepancy in selecting the candidate PvMATE responsible for PA monomer transport. The results revealed the differential accumulation of transcripts for the 4 candidate *PvMATEs* and raised the possibility that one of these 4 *PvMATEs* as a potential candidate for PA monomer transporter (**Figure 7**).

**Table 1:** Differential expression of candidate *PvMATE*s in pinto bean cultivars CDC Pintium and 1533-15 seed coat tissues (Duwadi et al., 2018).

Candidate	Gene ID	Transcript abundance (RPKM)		Fold change
		CDC Pintium	1533-15	
*PvMATE1*	*Phvul.001G103300*	93.244	174.647	0.534
*PvMATE6*	*Phvul.006G028700*	133.625	42.862	3.118
*PvMATE7*	*Phvul.007G034700*	608.294	10.902	55.797
*PvMATE8*	*Phvul.008G197000*	1765.641	374.705	4.712

**Figure 8:**
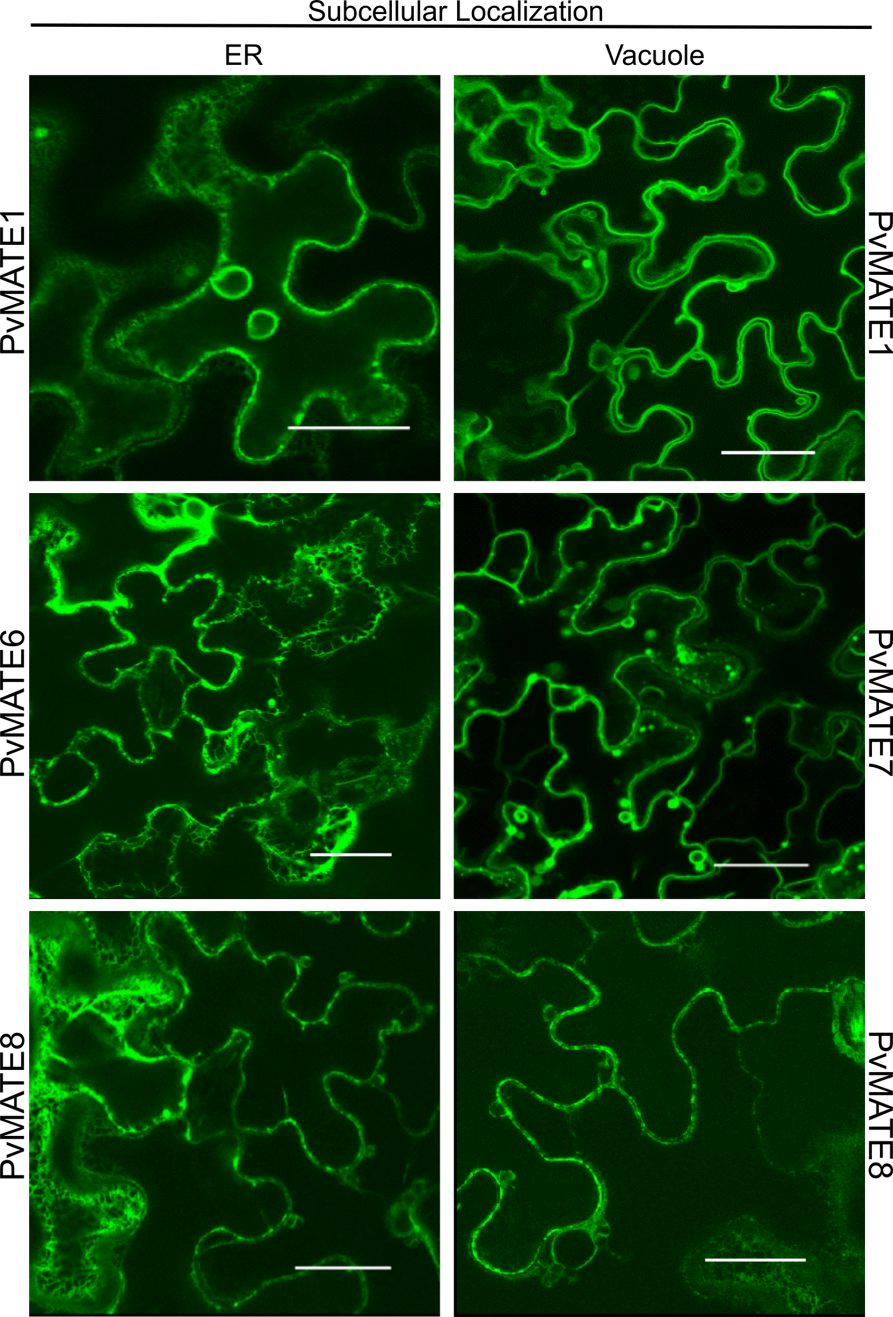
Subcellular localization of four selected PvMATE candidates. Each PvMATE was translationally fused upstream of the YFP reporter gene (visualized in green), co-transformed with *A. tumefacians* containing plasmids with organelle marker into *N. benthamiana via A. tumefacians*, and visualized in leaf epithelial cells by confocal microscopy. Merged signals are shown in **Supplementary Figure S5**. Scale: 30µm.

The protein that facilitates the transport of PA monomers inside the vacuole must be localized in the vacuolar membrane or tonoplast. The PA monomer transporter AtTT12 (Debeaujon et al., 2001; Marinova et al., 2007) and MtMATE1 (Zhao and Dixon, 2009) are located in the tonoplast. To investigate the subcellular location of candidate PvMATEs, the coding regions of each of the 4 selected *PvMATE*s was translationally fused with YFP, and transiently expressed in leaves of *N. benthamiana* followed by confocal microscopy. As shown in **Figure 8**, our result demonstrates that PvMATE1 and PvMATE8 localize in two subcellular compartments- tonoplast and endoplasmic reticulum (ER). PvMATE7 also localizes in the tonoplast whereas PvMATE6 was found in the ER only. Double layer membrane and florescent bulb projections are indicative to vacuole and tonoplast formation (Segami et al., 2014) while the network like structures around nucleus are indicative to the ER localization (Dastmalchi et al., 2016). Confirmation of the PvMATE localization in a specific subcellular compartment was performed by using the organelle-specific markers (**Supplementary Figure S5**). Since PvMATE6 was not found in the tonoplast, it was eliminated for further characterization. The localization of PvMATE8 in the tonoplast and itspresence in the C1-2 clade in the phylogenictic tree (**Figure 4**), provides a strong possibility of its involvement in the PA monomers transport.

### PvMATE8 is involved in PA accumulation

**Figure 9:**
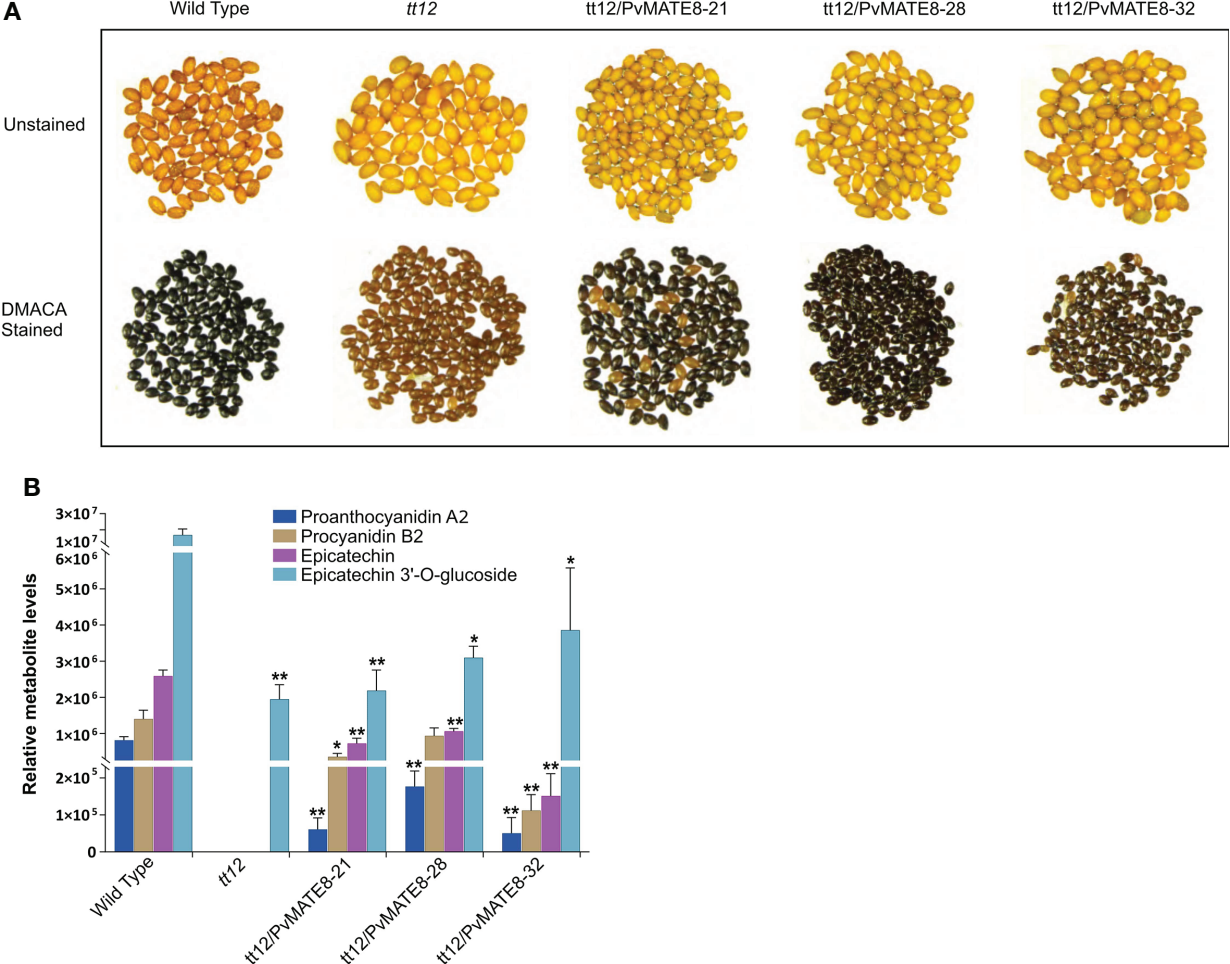
Genetic complementation of *tt12* by *PvMATE8* restores PA in Arabidopsis. **(A)** Unstained (top panel) and DMACA stained (bottom panel) seeds of wild type (Ws-2 ecotype), *tt12*, and three T2 complementation lines overexpressing PvMATE8-21, PvMATE8-28, PvMATE8-32 are shown. **(B)** LC-MS analysis to identify four target metabolites in corresponding Arabidopsis seeds, shown in **Figure 9A**. Each bar represents the relative abundance calculated from the peak area. Data are presented in SEM (n = 5). Significance was determined by one-way ANOVA (Dunnett’s *post hoc* test, alpha =0.05), *p<0.001, **p<0.00001 indicates a significant difference in relative abundance on each metabolite from the wild type.

Stable transformation for gene function characterization is a very reliable process, however, it is extremely difficult in common bean (Mukeshimana et al., 2013). Bean hairy root transformation serves as an alternative approach for bean researchers (Estrada-Navarrete et al., 2007). Since PA biosynthesis does not occur in the root tissues and is more specific for their function in the seed coat, we could not exploit the hairy root transformation for characterizing the PvMATEs. PA pathway is well characterized in Arabidopsis and the mutant lines offer a great platform for gene characterization for species such as common beans where plant transformation is extremely complicated. Thus we used Arabidopsis *tt12* mutant lines to perform a genetic complementation assay with the candidate *PvMATEs*. Mature wild type Arabidopsis seeds are dark brown while *tt12* mutants lack PA thus display a yellow phenotype. The tonoplast localized PvMATE candidates PvMATE1, PvMATE7 and PvMATE8 were overexpressed in Arabidopsis *tt12* and T2 seeds collected from multiple independent tt12/PvMATE transgenics were used for phenotyping. The results revealed that *PvMATE8* restored the wild type phenotype in the complementation lines, but PvMATE1 and PvMATE7 did not. **Figure 9A** shows the representative photographs of the unstained and DMACA stained wild type and *tt12* seeds with 3 T2 transgenic lines- tt12/PvMATE8 -21, tt12/PvMATE8 -28 and tt12/PvMATE8 -31. None of the 32 transgenic lines from tt12/PvMATE1 and tt12/PvMATE7 were able to rescue the mutant phenotype. Eight representative transgenic lines from tt12/PvMATE1, tt12/PvMATE7 and tt12/PvMATE8 stained with DMACA and photographed under the same condition are shown in **Supplementary Figure S6**.

Furthermore, we performed an LC-MS analysis to detect proanthocyanidin A2 (PA2) and procyanidin B2 (PB2), epicatechin and epicatechin 3’-O-glucoside (E3’G) in the seeds of *PvMATE8* overexpressed Arabidopsis lines and compared that with wild type and *tt12* mutants (**Table 2**; **Figure 9B**). Since E3’G is not commercially available, we used recombinant UGT72L1 in an enzymatic reaction with epicatechin to produce E3’G standard. The mass of the purified E3’G was confirmed (**Supplementary Figure S1**) prior to its use as a standard. PB2 is the predominant type of PA in Arabidopsis (Dixon et al., 2005). Mutant *tt12* seeds do not produce epicatechin and PAs (Marinova et al., 2007). Our results demonstrated that wild type seeds contained significantly higher levels of E3’G compared to aglycon epicatechin. Even though E3’G was found in *tt12* mutants, neither of the PA polymers nor epicatechin was detected in those mutant lines. However, PA polymers (PA2 and PB2) and epicatechin were restored in the complemented T2 transgenic lines- tt12/PvMATE8.

**Table 2:** Relative peak area of metabolites in wild type, *tt12* mutant and T2 Arabidopsis seeds over-expressing *PvMATE8*.

Metabolite	Wild Type	*tt12*	T2/PvMATE8-21	T2/PvMATE8-28	T2/PvMATE8-32
PA A2	8.60E+05	0.00E+00	6.11E+04	1.77E+05	5.03E+04
Procyanidin B2	1.44E+06	0.00E+00	4.03E+05	9.78E+05	1.12E+05
Epicatechin	2.61E+06	0.00E+00	7.72E+05	1.11E+06	1.51E+05
Epicatechin 3’-O-glucoside	1.68E+07	1.98E+06	2.22E+06	3.12E+06	3.87E+06

Data represent average of 5 biological replicates.

PA is a polymer of flavan-3-ols (e.g. catechin and epicatechin). The oligomers and polymers of PA differ in their structures in plants (Dixon et al., 2005). The type, number, position, stereochemistry and the modification of the flavan-3-ols (e.g. catechin and epicatechin) lead to the formation of different PA structures (Dixon et al., 2005; Lepiniec et al., 2006; Pang et al., 2007). MATE transporters of clade C1-2 were identified in other plants to transport specifically the E3’G into the vacuole (Marinova et al., 2007; Zhao and Dixon, 2009). Our results suggest that epicatechin accumulates in the vacuole after de-glycosylation of E3’G. Overexpression of *PvMATE8* in mutant *tt12* restored the wild type phenotype in the complementation lines by rescuing mutant phenotype. Accumulation of PA in the tt12/PvMATE8 lines provides a strong evidence that PvMATE8 is a transporter of E3’G.

Since Arabidopsis does not produce catechins (Tanner et al., 2003), the role of PvMATE8 in the transport of catechin could not be evaluated. The discrepancies observed between the wild type and the complementation lines in the levels of PA2 and E3’G could be due to the fact that common bean PvMATE8 transporter may not function as efficiently as TT12 in Arabidopsis. It was interesting to find E3’G in all the lines including *tt12*. An efficient transport of E3’G into the vacuole possibly induces the production of epicatechin. When the basal level of E3’G exceeds a threshold, gives a signal for epicatechin synthesis. DFR and ANR are the two terminal enzymes for epicatechin biosynthesis. Expression of both *DFR* and *ANR* is regulated by a MYB transcription factor, TT2 in Arabidopsis (Devic et al., 1999; Nesi et al., 2001; Debeaujon et al., 2003). Zhao and Dixon (2009) showed that *TT12* transcripts are not present in Arabidopsis *tt2* lines. These reports together with our present finding strongly suggest a feedback mechanism for the regulation of PA biosynthesis and accumulation in common bean.

## Conclusions

This study covers two key aspects- 1) a global analysis of PvMATEs; and 2) identification of PvMATE8 as a transporter of PA monomers in *P. vulgaris*. Comprehensive analysis of MATEs in common bean genome identified 59 PvMATEs in landrace G19833 along with their homologues in pinto UI111 and grouped them into their potential substrate-specific categories. Using the gene expression analysis, subcellular localization, complementation assay and metabolite analysis, we confirmed that PvMATE8 is the transporter of PA monomer in common bean.

## Data availability statement

The original contributions presented in the study are included in the article/**Supplementary Material**. Further inquiries can be directed to the corresponding author.

## Author contributions

NI performed *in silico* analysis, subcellular localization, PA staining and assays and wrote the first draft manuscript; KD, performed qPCR analysis and gene cloning; LC did complementation assays, TM and AP synthesized E3`G, FM contributed to E3`G experimental design and manuscript preparation, and SD conceived and designed the experiments, supervised all aspects of the project, and prepared the final draft manuscript. All authors contributed to the article and approved the submitted version.

## Funding

This research was supported by Agriculture and Agri-Food Canada’s Abase grants (J-000149 and J-001331) and a Natural Sciences and Engineering Research Council of Canada’s Discovery Grant (385922-2011 RGPIN) to SD.

## Acknowledgments

The authors thank Dr. Justin Renaud (AAFC) for help with LCMS analysis and Alex Molnar, Kuflom Kuflu and Praveen Khatri for technical assistance.

## Conflict of interest

The authors declare that the research was conducted in the absence of any commercial or financial relationships that could be construed as a potential conflict of interest.

## Publisher’s note

All claims expressed in this article are solely those of the authors and do not necessarily represent those of their affiliated organizations, or those of the publisher, the editors and the reviewers. Any product that may be evaluated in this article, or claim that may be made by its manufacturer, is not guaranteed or endorsed by the publisher.

## Supplementary material

Supplementary Figure 1 Chromatogram of synthesized Epicatechin 3`-O-glucoside (red) from standard Epicatechin (blue). UV Spectrum (absorbance at 224 nM) is shown on the inset.

Supplementary Figure 2 Protein sequence alignment of two PvMATE pairs between G19833 and pinto UI111- Phvul.004G152301 and PvUI111.04G149500 **(A)**, Phvul.004G152601 and PvUI111.04G149700 (B). Alignment coverage of Phvul.004G152301 and Phvul.004G152601 with the C- terminal halves pinto UI111 targets indicates a possible error in the sequence assembly.

Supplementary Figure 3 Genomic distribution of *PvMATE* genes in *P. vulgaris* cultivar pinto UI111 chromosomes. The chromosome numbers are indicated above each chromosome and drawn to scale in megabase pairs (Mbp). The chromosome size is indicated by its relative length using the information from Phytozome 13.

Supplementary Figure 4 Logo representation of the conserved motifs in PvMATEs shown in . The X-axis indicates the translated sequence length in amino acids, Y-axis indicates the bit score.

Supplementary Figure 5 Subcellular localization of 4 selected PvMATE candidates. Each PvMATE was translationally fused upstream of the YFP reporter gene, co-transformed with respective organelle localization markers (vacuole and ER)-fused CFP into *N. benthamiana via A. tumefacians*, and visualized in leaf epithelial cells by confocal microscopy. Merged signals were collected by sequential scanning of YFP and CFP channels. Scale: 30 µm.

Supplementary Figure 6 DMACA stained seeds in 8 individual T2 lines overexpressed with PvMATE1, PvMATE7 and PvMATE8. The picture was taken in normal light after 36 h of staining.
