# Supplementary figures and images for: Global analysis of common bean multidrug and toxic compound extrusion transporters (PvMATEs): PvMATE8 and pinto bean seed coat darkening
Source: Front Plant Sci. 2022 Nov 10;13:1046597. doi: 10.3389/fpls.2022.1046597 (PMC9686396; doi:10.3389/fpls.2022.1046597)

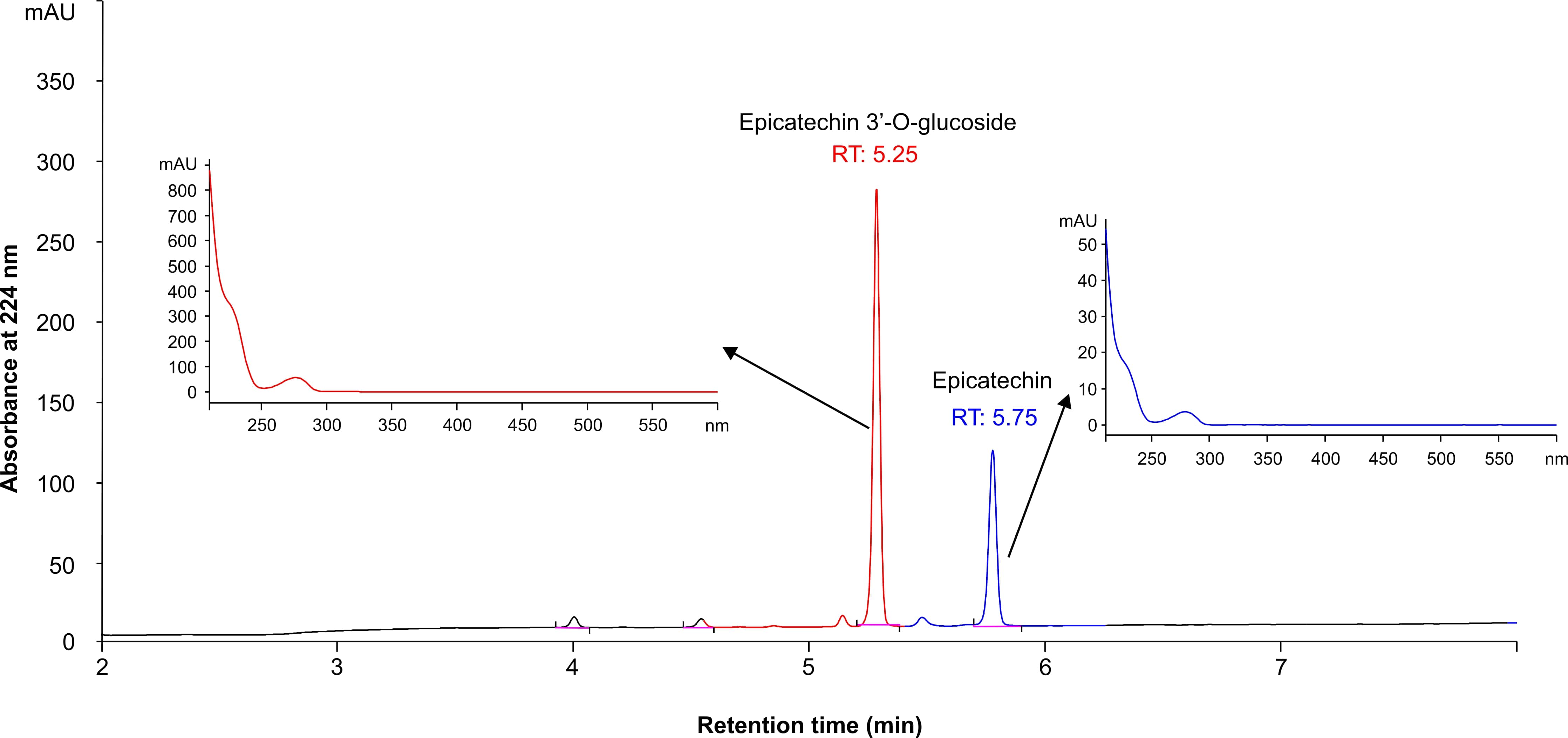

Supplement: Supplementary Figure 1 — Chromatogram of synthesized Epicatechin 3`-O-glucoside (red) from standard Epicatechin (blue). UV Spectrum (absorbance at 224 nM) is shown on the inset. [file Image_1.jpeg]

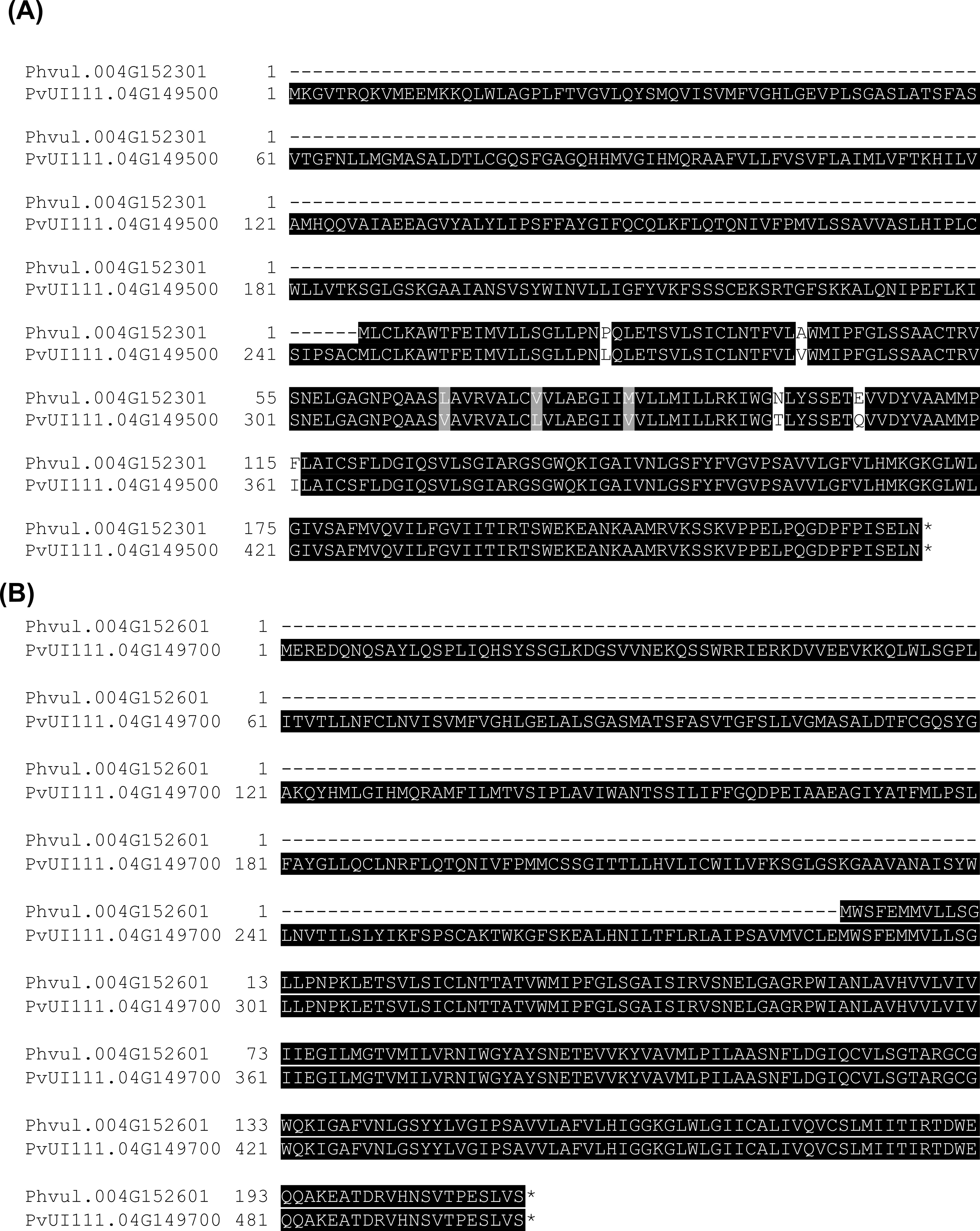

Supplement: Supplementary Figure 2 — Protein sequence alignment of two PvMATE pairs between G19833 and pinto UI111- Phvul.004G152301 and PvUI111.04G149500 (A), Phvul.004G152601 and PvUI111.04G149700 (B). Alignment coverage of Phvul.004G152301 and Phvul.004G152601 with the C- terminal halves pinto UI111 targets indicates a possible error in the sequence assembly. [file Image_2.jpeg]

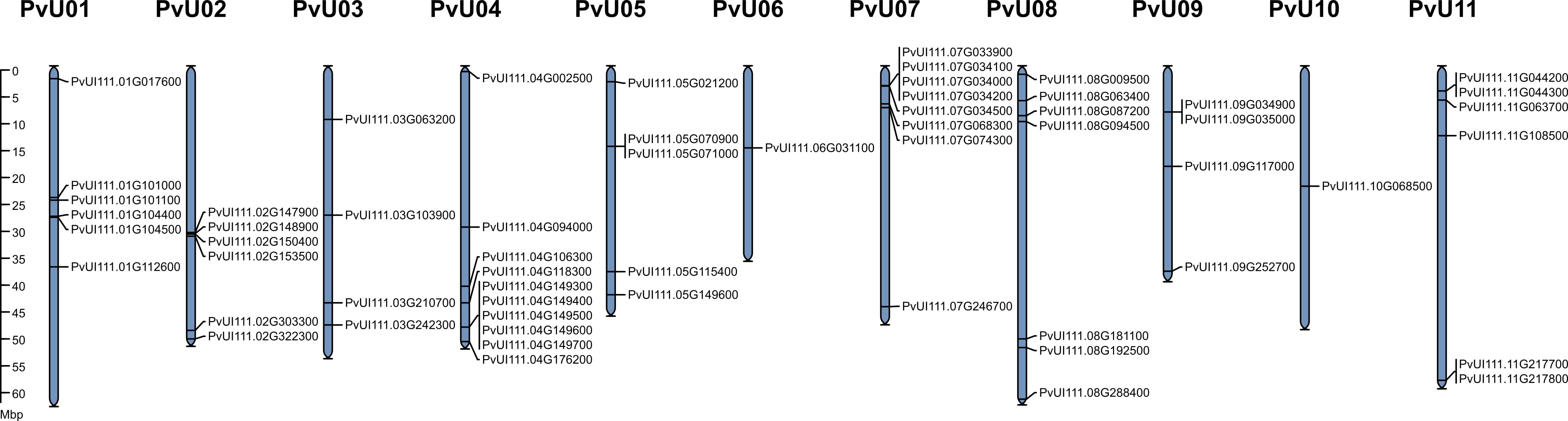

Supplement: Supplementary Figure 3 — Genomic distribution of PvMATE genes in P. vulgaris cultivar pinto UI111 chromosomes. The chromosome numbers are indicated above each chromosome and drawn to scale in megabase pairs (Mbp). The chromosome size is indicated by its relative length using the information from Phytozome 13. [file Image_3.jpeg]

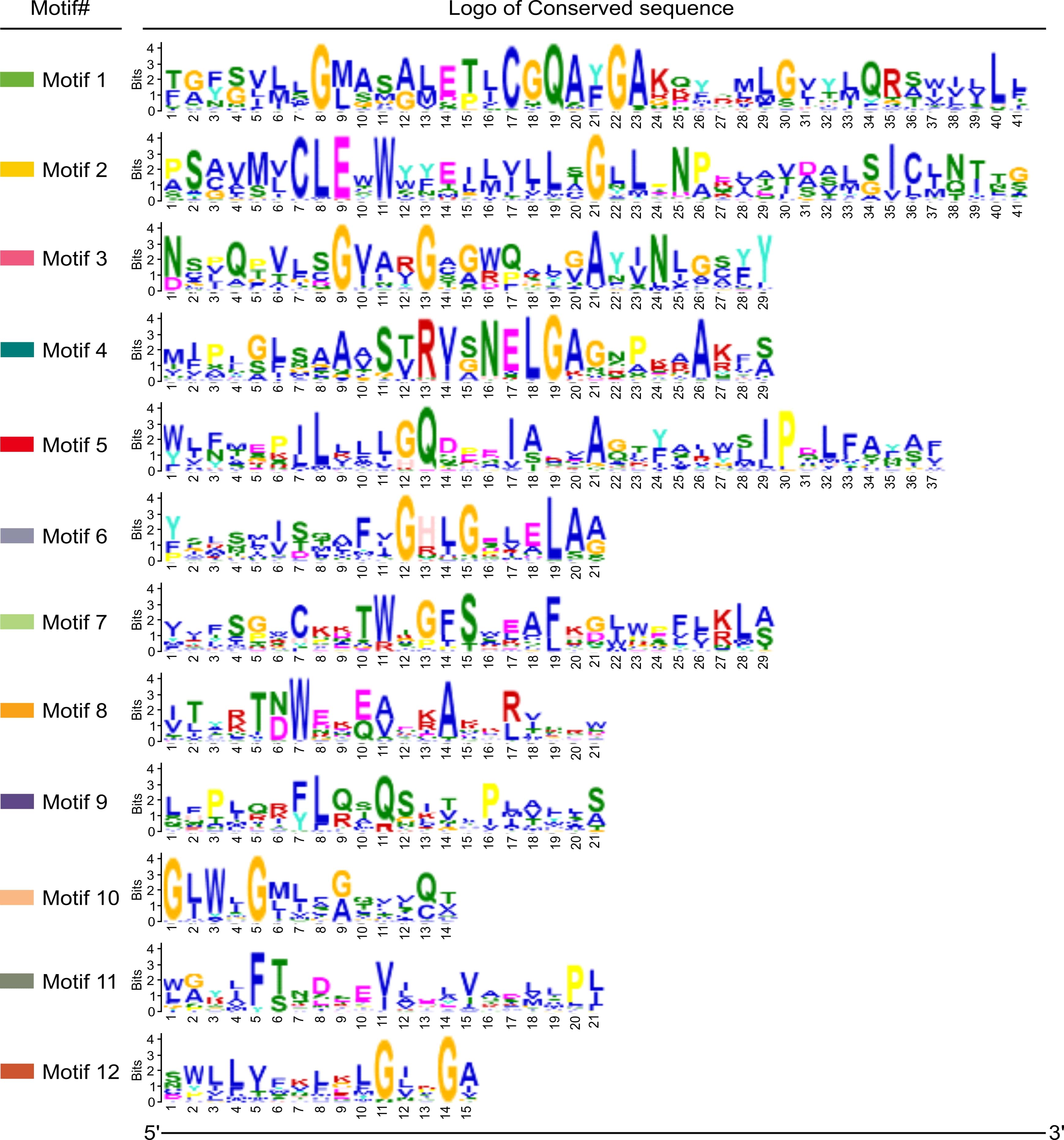

Supplement: Supplementary Figure 4 — Logo representation of the conserved motifs in PvMATEs shown in . The X-axis indicates the translated sequence length in amino acids, Y-axis indicates the bit score. [file Image_4.jpeg]

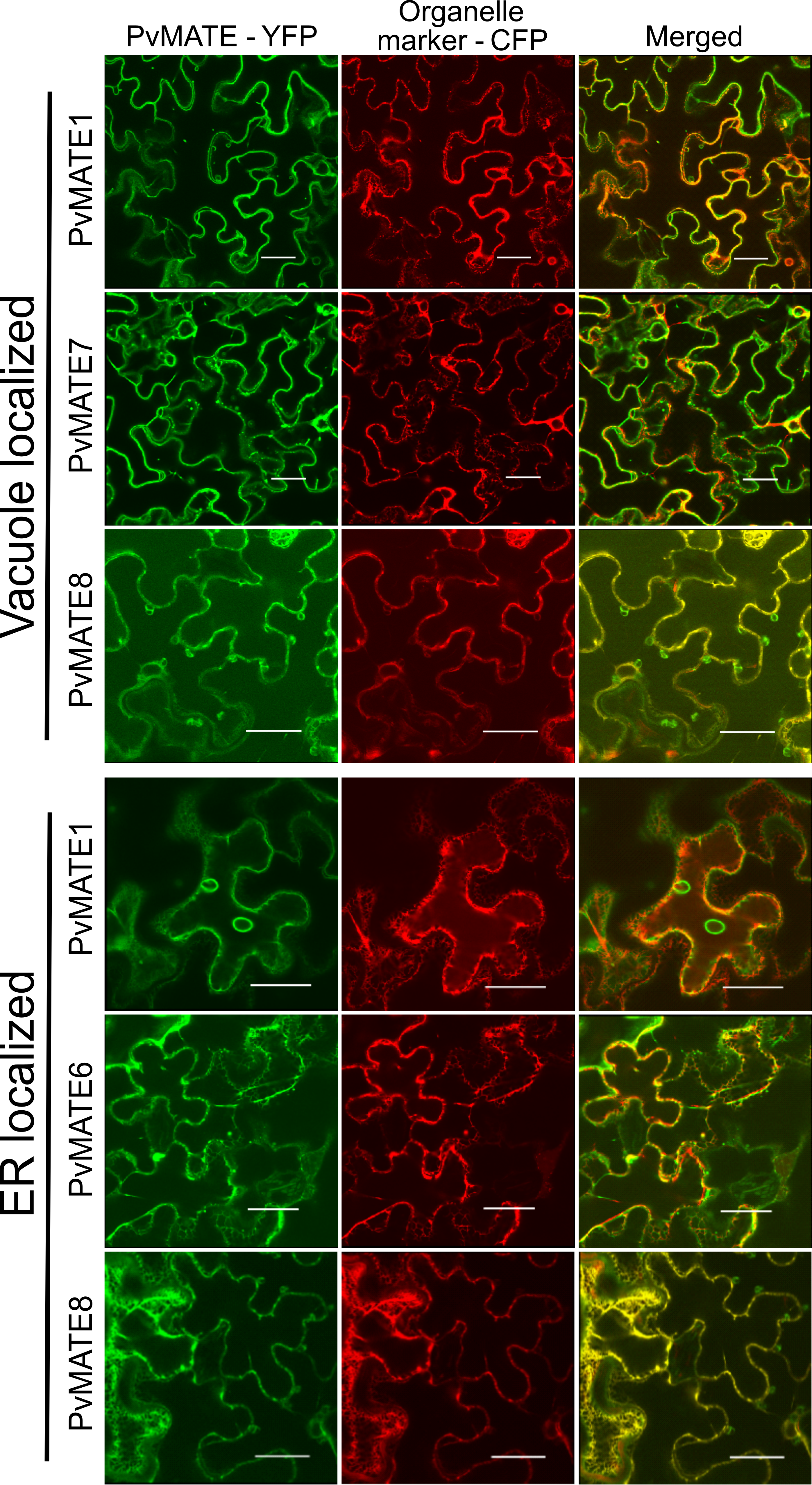

Supplement: Supplementary Figure 5 — Subcellular localization of 4 selected PvMATE candidates. Each PvMATE was translationally fused upstream of the YFP reporter gene, co-transformed with respective organelle localization markers (vacuole and ER)-fused CFP into N. benthamiana via A. tumefacians, and visualized in leaf epithelial cells by confocal microscopy. Merged signals were collected by sequential scanning of YFP and CFP channels. Scale: 30 µm. [file Image_5.jpeg]

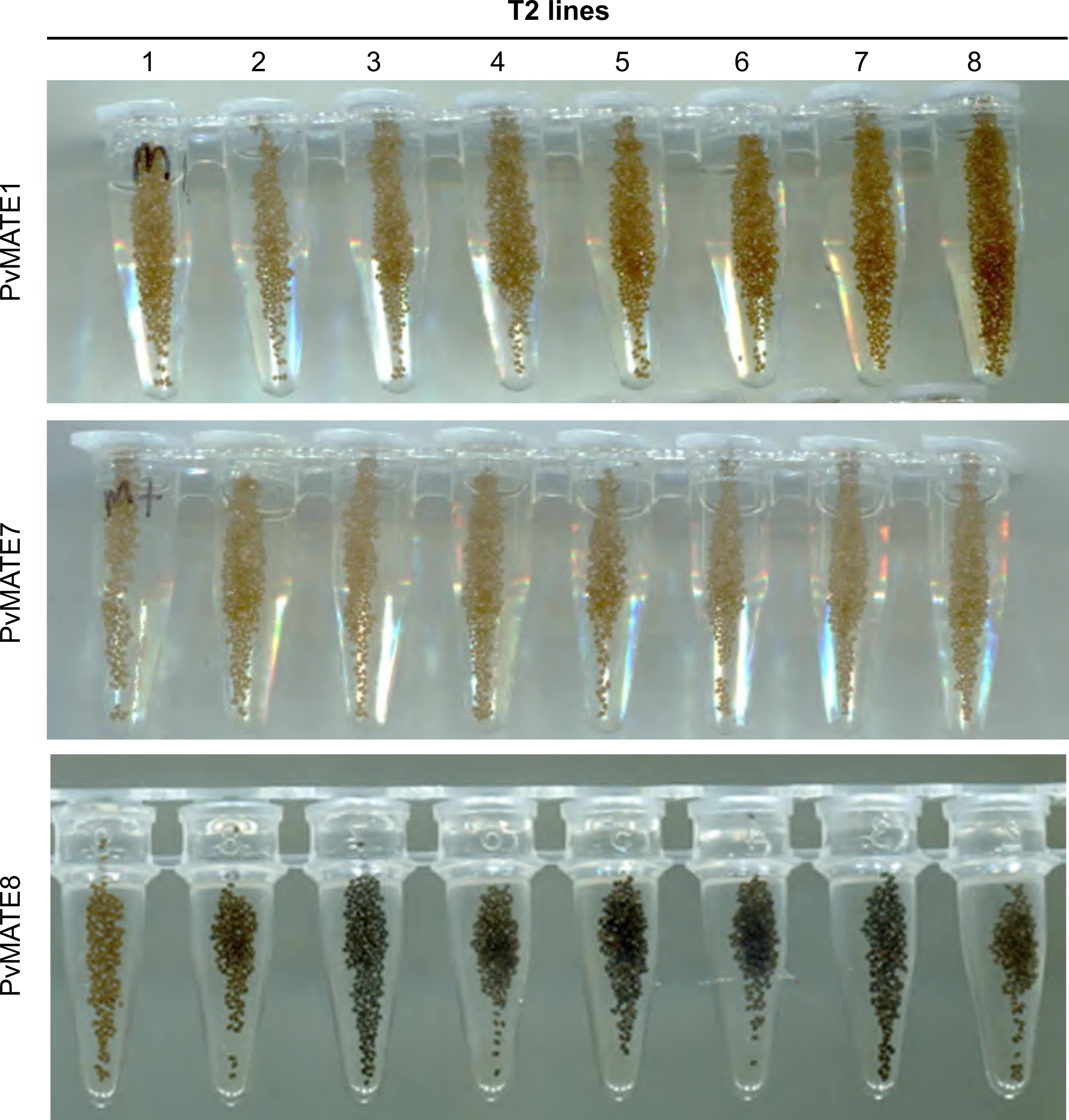

Supplement: Supplementary Figure 6 — DMACA stained seeds in 8 individual T2 lines overexpressed with PvMATE1, PvMATE7 and PvMATE8. The picture was taken in normal light after 36 h of staining. [file Image_6.jpeg]
