# Supplementary material for: Global analysis of common bean multidrug and toxic compound extrusion transporters (PvMATEs): PvMATE8 and pinto bean seed coat darkening
Source: Front Plant Sci. 2022 Nov 10;13:1046597. doi: 10.3389/fpls.2022.1046597 (PMC9686396; doi:10.3389/fpls.2022.1046597)
Supplement: Supplementary file 7 [file DataSheet_1.zip › Table s6.docx]

**Table S6**: List of primers used in gene cloning, qPCR and genotyping

| **Primer name** | **Sequence 5' to 3'** | **Purpose** | **Accession** |
| --- | --- | --- | --- |
| PvMATE1F | GGGGACAAGTTTGTACAAAAAAGCAGGCTCCATGGAG GGAGATCAAGAGG | ORF cloning | Phvul.001G103300.1 (*PvMATE1*) |
| PvMATE1R | GGGGACCACTTTGTACAAGAAAGCTGGGTCATT ATTTGATGTAATTCTTTCAGGATC |  |  |
| PvMATE1qF | AGGTGGTCGAAGGAGAGTGC | qPCR |  |
| PvMATE1qR | CGAAGCAAATTGGCATTCTTACGAGT |  |  |
| PvMATE6F | GGGGACAAGTTTGTACAAAAAAGCAGGCTCC ATGAACTCAGAATCAGGTTACCAACC | ORF cloning | Phvul.006G028700.1 (*PvMATE6*) |
| PvMATE6R | GGGGACCACTTTGTACAAGAAAGCTGGGTCGTA GTTGGCTATCAAGTGATCTAGGG |  |  |
| PvMATE6qF | TGGAGGTAACAAGCATGAACTC | qPCR |  |
| PvMATE6qR | TTGCTTGGGAGTCAAGGCTC |  |  |
| PvMATE7F | GGGGACAAGTTTGTACAAAAAAGCAGGCTCC ATGGAGGAAAGTCTACTACAGAAG | ORF cloning | Phvul.007G034700.1 (*PvMATE7*) |
| PvMATE7R | GGGGACCACTTTGTACAAGAAAGCTGGGTCTAC CAATATATTTTCTGCTGACAAT |  |  |
| PvMATE7qF | AAACATGGAGGAAAGTCTACTA | qPCR |  |
| PvMATE7qR | CAGCTAGGGAAATGGCTAAG |  |  |
| PvMATE8F | GGGGACAAGTTTGTACAAAAAAGCAGGCTCC ATGGGCTCCGTGGACTTTAATGAC | ORF cloning | Phvul.008G197000.1 (*PvMATE8*) |
| PvMATE8R | GGGGACCACTTTGTACAAGAAAGCTGGGTCTGA GTCAGTAACCAGTTCATCTAAGG |  |  |
| PvMATE8qF | CCTCACTCCATGCACTCCAAC | qPCR |  |
| PvMATE8qR | GCCTTGACTCCCACAGAACAA |  |  |
| PvUbiqF | ACAGCTGGAGGATGAAAGGA | Reference for qPCR;  *P. vulgaris* Ubiquitin | Phvul.007G052600.1 (Reference gene for *P. vulagris*) |
| PvUbiqR | GTCCGAACTCTCCACCTCAA |  |  |
| LPCS5740 | CAGATAACTGAACGGTCTTCGCC | Left primer for genotypying *attt12-1* | AT3G59030.1 (*TT12*) |
| RPCS5740 | AAGGATTGGACCCGAGTACCAGTAG | Right primer for genotyping *attt12-1* |  |
