# Supplementary material for: Global analysis of common bean multidrug and toxic compound extrusion transporters (PvMATEs): PvMATE8 and pinto bean seed coat darkening
Source: Front Plant Sci. 2022 Nov 10;13:1046597. doi: 10.3389/fpls.2022.1046597 (PMC9686396; doi:10.3389/fpls.2022.1046597)
Supplement: Supplementary file 7 [file DataSheet_1.zip › Table s7.docx]

**Table S7:** Standards used in metabolite analysis using LC-MS

| **Metabolite** | ***mz*** | **rt** | **Company, CAS#, Lot#** |
| --- | --- | --- | --- |
| Proanthocyanidin A2 | 575.12 | 2.61 | BOC Science, USA, CAS# 41743-41-3, LOT# EISV1808202 |
| Procyanidin B2 | 577.14 | 2.37 | BOC science, USA, CAS# 29106-49-8, LOT# EISV18V01101 |
| Epicatechin | 289.07 | 2.44 | INDOFINE Chemical Company, USA, CAS# 490-46-0, LOT# 1305326 |
| Epicatechin 3’-O-glucoside | 451.12 | 2.32 | Synthesized in-house |

*mz*, mass to charge ratio; rt, retention time
